# Accelerated Bioprocess Development of Endopolygalacturonase-Production with *Saccharomyces cerevisiae* Using Multivariate Prediction in a 48 Mini-Bioreactor Automated Platform

**DOI:** 10.3390/bioengineering5040101

**Published:** 2018-11-21

**Authors:** Annina Sawatzki, Sebastian Hans, Harini Narayanan, Benjamin Haby, Niels Krausch, Michael Sokolov, Florian Glauche, Sebastian L. Riedel, Peter Neubauer, Mariano Nicolas Cruz Bournazou

**Affiliations:** 1 Department of Bioprocess Engineering, Department of Biotechnology, Technische Universität Berlin, Ackerstr. 71-76, ACK24, D-13355 Berlin, Germany; a.sawatzki@campus.tu-berlin.de (A.S.); sebastian.hans@tu-berlin.de (S.H.); benjamin.haby@tu-berlin.de (B.H.); n.krausch@campus.tu-berlin.de (N.K.); florian.glauche@tu-berlin.de (F.G.); riedel@tu-berlin.de (S.L.R.); peter.neubauer@tu-berlin.de (P.N.); 2 ETH Zürich, Rämistrasse 101, CH-8092 Zurich, Switzerland; nharini@chem.ethz.ch (H.N.); m.sokolov@datahow.ch (M.S.); 3 DataHow AG, c/o ETH Zürich, HCl, F137, Vladimir-Prelog-Weg 1, CH-8093 Zurich, Switzerland

**Keywords:** mini-bioreactors, high throughput bioprocess development, laboratory automation, biomanufacturing, digitalization, multivariate analysis, dynamical bioprocesses

## Abstract

Mini-bioreactor systems enabling automatized operation of numerous parallel cultivations are a promising alternative to accelerate and optimize bioprocess development allowing for sophisticated cultivation experiments in high throughput. These include fed-batch and continuous cultivations with multiple options of process control and sample analysis which deliver valuable screening tools for industrial production. However, the model-based methods needed to operate these robotic facilities efficiently considering the complexity of biological processes are missing. We present an automated experiment facility that integrates online data handling, visualization and treatment using multivariate analysis approaches to design and operate dynamical experimental campaigns in up to 48 mini-bioreactors (8–12 mL) in parallel. In this study, the characterization of *Saccharomyces cerevisiae* AH22 secreting recombinant endopolygalacturonase is performed, running and comparing 16 experimental conditions in triplicate. Data-driven multivariate methods were developed to allow for fast, automated decision making as well as online predictive data analysis regarding endopolygalacturonase production. Using dynamic process information, a cultivation with abnormal behavior could be detected by principal component analysis as well as two clusters of similarly behaving cultivations, later classified according to the feeding rate. By decision tree analysis, cultivation conditions leading to an optimal recombinant product formation could be identified automatically. The developed method is easily adaptable to different strains and cultivation strategies, and suitable for automatized process development reducing the experimental times and costs.

## 1. Introduction

In biomanufacturing, processes developed in R&D often suffer setbacks during transfer to industrial production [1]. Hence, a more consistent bioprocess development from the screening to the production phase through a higher throughput while maintaining a high explanatory power of the experiments is needed [1,2,3]. To reduce the risk of potential failures during scale-up, small-scale screening systems should mimic large-scale conditions. On the other hand, since high throughput (HT) systems focus on increase of the number of parallel experiments, a trade-off must be met sacrificing the sophistication of cultivations’ monitoring and controls and its relevance for industrial scale.

Mini-bioreactor (MBR) systems, which have been described as scalable to benchtop bioreactors [4], are an effort to fill this gap. In comparison to lab-scale bioreactors, MBRs allow for a higher experimental throughput—e.g., the fast screening of large strain libraries [5] or a great number of experimental conditions [6,7]—while still enabling the implementation of large-scale process conditions such as feeding, closed loop controls and techniques for scale-down experiments [8,9]. Additionally, their integration into liquid handling robots allows for execution of multiple manipulations in parallel. Pre-defined additions of feed stock or programmed pH control around a given set-point is possible, as well as automated sampling and at-line analysis of the samples by integration of laboratory devices such as spectrophotometers [10,11]. Finally, due to the high number of parallel experiments, multiple experimental set-ups can be tested including replicates, which increases the reliability and transferability of the generated data for scale-up purposes. 

**Figure 1:**
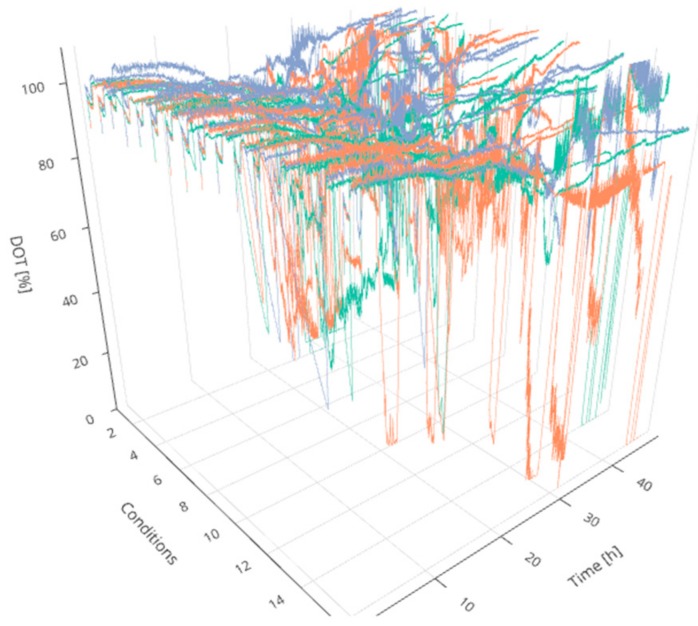
3D representation of 48 experiments. The dissolved oxygen tension (DOT) profiles are ordered in 16 groups indicating triplicate experiments over the time of cultivation. The colors have been added for visibility. Interactive versions of the plots can be found online [19].

Following the scopes of industry 4.0 [2,12], smart digital solutions should not only be used to support the process development procedure but to operate sophisticated robotic facilities during development [2]. For this purpose, four technical aspects are crucial: (i) centralized online data storage and handling for real-time monitoring and visualization, (ii) mathematical methods for data analysis to support decisions during operation, (iii) a full integration of all operated devices, sensors and stakeholders in one accessible and consistently updated digital platform, and (iv) an efficient workflow with proper scheduling assistance and resource availability. Regarding experiments in MBR systems, traditional process control methods face additional challenges since a control strategy for various cultivations simultaneously (here 48) is required. Even a comparative evaluation of the cultures in real-time is difficult due to the considerable amount of manipulated and controlled process parameters as well as the dynamically evolving process variables (see Figure 1).

Today, we lack the computer aided tools to design and operate efficient experiments in such highly parallelized systems. Currently, there are two main approaches that are being followed to solve these issues. Firstly, automation of experimental facilities towards smart platforms [13] and sequential designs, by which experimental data is processed by algorithms to design the next experiments [14,15,16]. These methods use either static models (e.g., regression models) or a restricted level of feed-back operation. In other words, the information generated during the experiment is not exploited to its full potential aiming to a smart and optimal operation of the robots. Secondly, model-based optimal experimental design methods [17,18] are being developed to compute and perform optimal operation strategies in a closed loop environment. Unfortunately, such advanced methods need a thorough understanding of the culture and some kind of mechanistic models (e.g., macro-kinetic growth models), which especially in the development stages are difficult to obtain.

There exist very good examples of the requirements and potential of advanced operation strategies for parallel cultivations in MBR systems: The influence of two aeration concepts was tested in 24 MBR cultivations in the ambr 15f system (Sartorius Stedim Biotech, Royston, UK) and compared to the large scale [20]. Biomass was sampled regularly, however sampling for offline analyses occurred at low frequency and the analyses were not automated. Screenings in 48 MBRs in the bioREACTOR 48 fermentation system (2mag AG, Munich, Germany) were performed regarding the performance of recombinant *E. coli* strains with product determination only at the end of the fermentation [5] or regarding recombinant *Bacillus subtilis* strains with samplings every 24 h as well as pH measurement every 30 min [21]. Hortsch et al. [22] performed growth media comparison for *E. coli* cultivations in 48 MBRs with regular biomass determination but limited offline sampling. 

However, to the best of the authors’ knowledge, the HT platforms using MBRs described above have not yet reached important milestones in HT bioprocess development as are:

(i) fully automated long-term cultivations (at least 48 h) with adaptive input experimental design and operation of 48 fed-batch cultivations in parallel MBRs and,
(ii) an automated online data evaluation, visualization and robot operation with limited a priori knowledge using multivariate statistical methods.

We tackle these challenges and demonstrate the advantages of our developed methods in this study. For this, an experimental run of 48 MBR fed-batch cultivations, integrating efficient parallel operation routines, control of temperature, pH, and aeration for all MBRs, as well as multivariate methods for analysis of online and at-line data generated with advanced sensor technologies is presented. Data-driven statistical methods enable fast, automated and parallel decision making and online predictive data analysis for each cultivation as well as the campaign as a whole. A digital platform facilitates central information storing, accessible to process engineers and process models.

As a case study, *S. cerevisiae* AH22 secreting a pectinase from *Aspergillus niger* [23] serves as an example organism for development of recombinant production processes. However, the developed methods are not specific to the strain or the cultivation conditions and can be used in a wide range of screening experiments. *S. cerevisiae* has been a production host for recombinant products such as insulin, Hepatitis B vaccine as well as growth hormones since the 1980s [24] and is still used for a wide range of products, e.g., therapeutic compounds [25], the antioxidant resveratrol [26], and also for production of enzymes in the food industry, for instance pectinases. Besides rapid growth and a well-characterized genome, *S. cerevisiae* offers greater robustness in industrial processes, GRAS (Generally Regarded As Safe)-status, secretion of very few endogenous proteins and direction of recombinant proteins correctly-folded into the culture supernatant, which simplifies purification [23,25,27,28]. Yeasts are capable of posttranslational modifications, even though the glycosylation pattern is high in mannose [24] and genetic engineering of the secretory pathways in *S. cerevisiae* is difficult [29,30]. HT systems can contribute to an accelerated strain characterization and selection. Automated treatment for competence and transformation in high-throughput manner as described in [31,32] will help in constructing heterologous yeast strains producing desired target molecules. 

To achieve an optimal production of recombinant products, important factors to consider during the process are the maximization of the biomass and the cultivation time so to improve the product yield. Additionally, the optimal growth rate for protein production, the reduction of ethanol production and similar by-products due to overflow conditions should be considered. *S. cerevisiae* produces ethanol not only during oxygen limitation due to the Pasteur effect, but also aerobically when the substrate concentrations exceeds a critical strain-specific value [33] through the Crabtree effect or overflow metabolism caused by a maximum in the respiration capacity [34,35]. Ethanol is resorbed by the cells when the preferred substrate—namely fermentable sugars such as glucose—is exhausted. However, growth on ethanol is slower than growth on glucose, while increasing the oxygen demand [33,36].

Given that during strain development, parameters such as the maximal growth rate or optimal production conditions are not well known, design of experiments (DoE) is applied to statistically determine which experimental input influences the studied system in a way that leads to the most informative experiments and should guide process optimization through experiments sequentially approaching the optimal target conditions. By these methods experiments not resulting in knowledge gain are avoided, thereby minimizing the time and resources needed for process development. Finally, this work is a step further to close the gap between data-driven statistical models and knowledge-driven deterministic models by combining them in so-called “hybrid models”, which can be flexibly defined given the available process knowledge and data [37,38,39].

## 2. Materials and Methods

### 2.1. Strain and Culture Storage

Cultivations were performed using *S. cerevisiae* AH22 (leu2-1, leu2-112, his4-519, can1, cir^+^, mating type a), harboring the plasmid pPG6 constructed for the heterologous expression of endopolygalacturonase (EPG) from *A. niger* [23,40]. The recombinant strain pPG6 M27 showing improved pectin hydrolysis was used previously [16]. Stock cultures were stored in 1 mL aliquots with 20% glycerol at −80 °C.

### 2.2. Media

For all cultivations modified WMVIII minimal medium containing NH_4_H_2_PO_4_ 0.25 g L^−1^, NH_4_Cl 5.48 g L^−1^, MgCl_2_·6H_2_O 0.25 g L^−1^, CaCl_2_·2H_2_O 0.1 g L^−1^, KH_2_PO_4_ 2.0 g L^−1^, MgSO_4_·7H_2_O 0.55 g L^−1^, myo-inositol 75 mg L^−1^, EDTA 11.69 mg L^−1^, ZnSO_4_·7H_2_O 1.75mg L^−1^, FeSO_4_·7H_2_O 0.5 mg L^−1^, CuSO_4_·5H_2_O 0.1 mg L^−1^, MnCl_2_·4H_2_O 0.1 mg L^−1^, Na_2_MoO_4_ 2H_2_O 0.1 mg L^−1^, nicotinic acid 10 mg L^−1^, pyridoxin-HCl 25 mg L^−1^, thiamine-HCl 10 mg L^−1^, biotin 2.5 mg L^−1^, calcium pantothenate 50 mg L^−1^, histidine 100 mg L^−1^ and 0.01% Antifoam 204 (Sigma Aldrich, St. Louis, Missouri, USA) was used [16]. The WMVIII medium was complemented with glucose in different concentrations as a carbon source.

### 2.3. Precultures

The first preculture was inoculated from one cryo vial into 25 mL modified WMVIII medium, which was supplemented with 40 g L^−1^ glucose, 1.5 g L^−1^ sodium glutamate, and buffered with 5% citrate-phosphate buffer (pH 6) in a 100 mL UltraYield^TM^ flask covered with AirOtop^TM^ Enhanced Seal (both Thomson Instrument Co., Oceanside, California, USA). The preculture was incubated for 24 h at 30 °C and 180 rpm in an orbital shaker (Lab-Therm LT-X, Adolf Kühner AG, Basel, Switzerland; 50 mm amplitude).

A second preculture was inoculated from the first preculture to an optical density at 600 nm (OD_600_) of 0.3 into 100 mL medium in a 500 mL flask and cultivated under the same conditions as the first preculture for 24 h.

### 2.4. Main Culture

The main culture was inoculated from the second preculture to an OD_600_ of 0.3 into 300 mL of the modified WMVIII medium, which was supplemented with an initial substrate concentration of 20 g L^−1^, respectively 30 g L^−1^ glucose (Table 1). Under sterile conditions 10 mL of the inoculated medium were transferred into 48 mini-bioreactors of the pre-sterilized bioREACTOR 48 fermentation system (2mag AG, Munich, Germany). The set points of the circulation thermostat and the reflux cooler were 30 °C and 4 °C respectively. The cultures were aerated with 5 L min^−1^ of pressurized air and the stirrer speed was kept constant at 2400 rpm during the cultivation. Dissolved oxygen tension (DOT) and pH were measured by fluorescence sensors (PreSens Precision Sensing GmbH, Regensburg, Germany). The pH sensors were calibrated to a range of pH 5–8 and the DO sensors were adjusted with a two-point calibration under oxygen-free conditions by introducing nitrogen and under oxygen-saturation. The pH was controlled at 6.0 by titration of 3.5 M NH_3_ (one-sided control).

An initial batch phase of around 12 h was followed by a fed-batch phase with small bolus additions of a concentrated glucose stock solution every 5 min via the Freedom Evo liquid handling station (LHS) (Tecan, Männedorf, Switzerland), as described by Haby et al. [11]. The concentration of the feed stock was 100 g L^−1^ or 500 g L^−1^ for higher feed rates to lower the volume increase. 

After the cultivation, the final volume was measured to calculate the evaporation rate. For determination of the cell dry weight 1.5 mL of culture broth was collected in duplicates in pre-dried Eppendorf tubes and centrifuged. The supernatant was discarded, and the cell pellet was dried at 75 °C for more than 48 h.

### 2.5. Sampling

From every mini-bioreactor 250 µL of culture volume was taken column-wise with the 8-channel pipette of the Freedom Evo LHS and pipetted into 96 well microtiter plates containing 15 µL dried 2 M anhydrous NaOH per well to inhibit cell activity [11].

During the batch phase samples were drawn with a difference of five minutes between the individual columns, the at-line analysis (as described in Section 2.6) was performed in single determination without replicates. After beginning of the fed-batch phase samples were drawn every 20 min from the eight reactors of one column, consequently each mini-bioreactor is sampled every 2 h. Here, the at-line analysis was performed in double determination.

### 2.6. At-Line and Offline Analysis

As at-line values, OD_600_ and glucose concentration were determined as descripted by Haby et al. [11]. Additionally, the remaining supernatant was immediately sealed and stored at −20 °C for offline analysis. The ethanol concentration was determined offline using the Cedex Bio HT Analyzer (Roche Diagnostics International Ltd., Risch, Switzerland). The detection range of ethanol using the Cedex Analyzer Ethanol Bio HT Kit (ETOHB) is 0.5 to 10.1 g L^−1^. However, as the samples had to be diluted with an equal amount of deionized water prior to analysis due to their small volume, the lower detection limit was 1.0 g L^−1^. The volumetric enzymatic activity (EA) of EPG was determined by a colorimetric assay with 2-methyl-2-benzothiazolinonehydrazone (MBTH) in 96-well plate format using the Hamilton LHS as described elsewhere [16]. 

### 2.7. Experimental Design

The cultivations in the 48 mini-bioreactors were performed under 16 experimental conditions in triplicate based on a fractional factorial design. Two batch lengths—determined by an initial glucose concentration of either 20 g L^−1^ or 30 g L^−1^, three feed profiles—exponential, linear and constant, three feed rates—0.0875 h^−1^, 0.125 h^−1^ and 0.35 h^−1^, and an optional “hunger phase”—a period of 2 h after batch end, where no substrate was supplied—were applied. The full experimental plan is provided in Table 1.

### 2.8. Calculation of Feed Rates

The feed rates were calculated based on an initial feed rate *F*_0_:

(1) $$F_{0}=\frac{{µ}_{set}}{S_{i}*Y_{X/S}\;}\cdot X_{0}\cdot V_{0}$$

where µ*_set_* (h^−1^) represents the set-point of the specific growth rate—i.e., the feed rate which is given in Table 1, *S_in_* (g L^−1^) the glucose concentration in the feed, *Y_X/S_* (g g^−1^) the biomass yield coefficient, *V*_0_ (L) the starting volume and *X*_0_ (g L^−1^) the biomass concentration present at the start of the feeding in each mini-bioreactor (g L^−1^). *Y_X/S_* was estimated to 0.5 (g g^−1^) according to [40], determined for the same recombinant strain.

In the first feed phase with a duration of 12 h, the feed was increased either exponentially or linearly, or kept constant (see Table 1). The exponential feed F_exp_ (L h^−1^) was calculated from the initial feed rate F_0_, the set growth rate µ_set_ and the time *t* (h):

(2) $$F_{\exp}\;\left(t\right)=F_{0}\cdot e^{{µ}_{\mathrm{set}}\left(t\right)}$$

**Figure 2:**
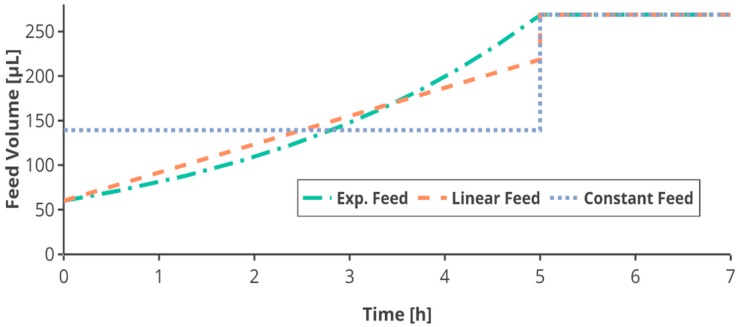
Schematic overview of the feed profiles. In the first feed phase exponential, linear or constant feed is applied according to the experimental plan, followed by a constant feed (here after 5 h) for all cultivations in the second feed phase. As the calculation of the linear and constant feed is based on the respective exponential feed, all cultivations receive the same amount of glucose during the cultivation, relative to the biomass concentration at the feed start. Interactive versions of the plots can be found online [19].

The linear and constant feed in the first feed phase were based on the total amount of glucose fed in a respective exponential feed (Appendix A). Therefore, all cultivations received the same amount of glucose after the first feed phase (Figure 2).

After 12 h the feed was switched to an equal constant feed for all feed profiles. To ensure the same feeding conditions, the feed rate applied in this feed phase was the same for all feed profiles corresponding to the same feed rate. As the feeding was applied using the semi-continuous method of small bolus additions every 5 min, the feed rates were discretized into pulses of 5-min intervals, adapted from Anane et al. [41]. Due to the limitation in total volume in the mini-bioreactor, the feed was limited to 30 µL per pulse, shortening the first fed-batch phase in some cultivations.

For calculation of the feed volumes as well as for the data processing and multivariate analysis, explained in the following, MATLAB 2016a, respectively 2017a was used (The MathWorks, Inc., Natick, Massachusetts, USA).

### 2.9. Data Processing

An SQL data structure was used for central data storage. The database can be accessed via a NET library with shared programming functions for the database communication currently running with Matlab^®^, Python™ and LabView^®^. For manual operation during the experiments, the database can be accessed via a General User Interface (GUI). It can be used to write set points and parameters manually to the database. The server-based communication between the two liquid handlers is assured by a SiLA (Standardization in Lab Automation) 2 protocol. The Tecan robot sends a SiLA2 Client command via the network to the SiLA2 Server on the Hamilton site, which triggers the start of the Hamilton script for at-line analysis.

The different feed and evaporation rates led to differing volumes in the mini-bioreactors. To simplify comparison, the OD_600_ values were normalized to the start volume of 10 mL considering the evaporation and the dilution by feed, base and medium addition. All other measurements were not normalized, so the concentrations might be affected by dilution. Regarding the specific EA, the volume difference had no influence and the values therefor did not have to be adapted.

The specific growth rate µ (h^−1^), the substrate consumption rate *q_S_* (g_substrate_ (g_biomass_ h)^−1^) and the specific product formation rate *q_P_* (U (g_biomass_ h)^−1^) were determined by the following equations:

(3) $$µ=\frac{\ln\left(X_{N,2}\right)-\ln\left(X_{N,1}\right)}{t_{2}-t_{1}}$$

(4) $$q_{S}=\frac{S_{2}-S_{1}}{(t_{2}-t_{1})\cdot X}$$

(5) $$q_{P}=\frac{EAv_{2}-EAv_{1}}{(t_{2}-t_{1)}}\cdot \frac{2}{X_{2}-X_{1}}$$

where *X* (g L^−1^) refers to the biomass concentration, *X_N_* (g L^−1^) refers to the normed biomass concentration, *S* (g L^−1^) to the substrate concentration, *t* (h) to the cultivation time and *EAv* (U mL^−1^) to the volumetric enzyme activity.

### 2.10. Multivariate Statistical Analysis

The data generated from the MBR was analyzed using statistical tools to aid process optimization based on the parallel experiments. The resulting three dimensional dataset (runs × variables × times) was unfolded in batch-wise manner [42] to obtain a table with the rows spanning the different experiments and the columns distinguishing different variables at different time points.

Firstly, for a dynamic comparison of the multivariate process behavior of the runs, batch-wise unfolded (BWU) principal component analysis (PCA) [43] using the biomass, glucose and ethanol concentration, pH, DOT, base additions, and volumes as input was performed. Score plots were used to identify abnormally behaving reactors and reactors that performed similarly. Statistically significant clusters were then identified in the score space automatically using k-means clustering algorithm [44]. Correspondingly, clusters were characterized based on the design of experiments using a classification tree analysis [45]. 

Additionally, for predicting the product characteristic based on the process behavior, a prediction model to estimate the EPG activity was developed using BWU historical partial least square regression (PLSR) model [46]. Thus, for instance, to build a prediction model for EPG activity at 22 h in addition to the design variables, the measured variables, namely biomass, glucose and ethanol concentration, pH, DOT and volumes until this time were used as input for the model. A variable selection routine to identify the most important variables and crucial measurement time points was implemented. Variables were added one at a time and the mean of the 10-fold cross validation [44] error was monitored to identify the variable combination providing the minimum mean root mean square error of cross validation (RMSECV). The RMSECV was computed regarding the total number of cross validations *N_runs_*, the predicted values *Y^pred^* and the observed values *Y^act^* using the following formula:

(6) $$\mathrm{RMSECV}=\sqrt{\frac{1}{N_{runs}}\overset{N_{runs}}{\underset{i=1}{\sum }}{\left(\frac{Y_{i}^{pred}-Y_{i}^{act}}{Y_{i}^{act}}\right)}^{2}}$$

Finally, the experimental conditions that could simultaneously minimize ethanol production and maximize EPG activity were identified using regression tree analysis [45]. Four independent regression trees were developed to predict the four targets, ethanol and EPG activity at 22 h and 35 h, using feeding profile, feed rate, initial substrate concentration and hunger phase data. The decision paths, i.e., the applied experimental conditions, leading to high EPG activity and low ethanol concentrations were identified in all the decision trees. The thus identified optimal experiments for production were evaluated for overlapping cultivation conditions, for example the same feeding rate or profile, to ascertain a suitable design.

## 3. Results

Screening and process development under fed-batch conditions in stirred MBRs enable strain phenotyping closer to industrial conditions while still reducing the experimental time and effort.

**Figure 3:**
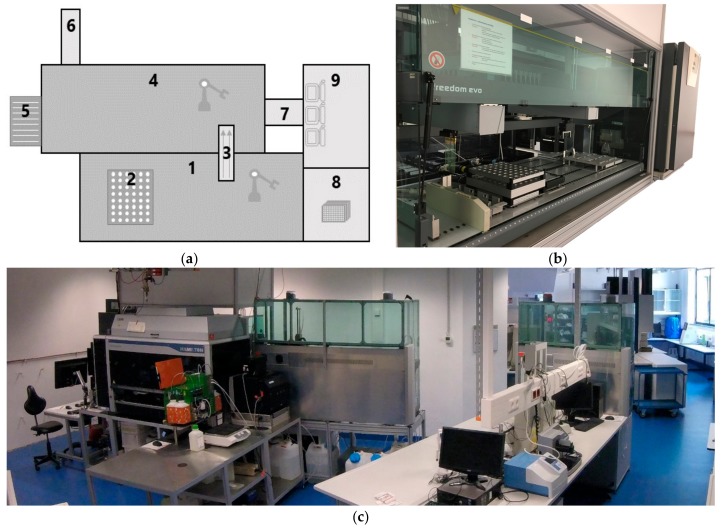
The robotic HT platform for cultivation in 48 MBRs and automated sampling and analysis. Shown are the Tecan LHS including the MBR system, the Hamilton LHS connected by a linear transfer unit. (**a**) Scheme of the robotic platform with 1-Tecan EVO 200, 2–bioREACTOR48 system, 3-Linear transfer unit, 4-Hamilton Microlab STAR, 5-SynergyTM MX Plate Reader, 6-MACS Quant Analyzer 10, 7-FAME incubator, 8-CytomatTM 6004 DR, 9-monitoring and control station, (**b**) robotic platform facing the Tecan Evo 200 with bioReactor48 system, (**c**) HT lab facing the Hamilton Microlab STAR.

To achieve this, a procedure for process control, sampling and analysis for 48 parallel mini-bioreactor cultivations in a HT platform (Figure 3) was developed. *S. cerevisiae* AH22 producing recombinant EPG was characterized in the milliliter scale regarding the best growth conditions for optimization of the final and specific product concentration. Advanced HT methods such as optimal scheduling and adaptation of the process operations, online data handling and automated sample analysis were applied.

Sixteen combinations of cultivation conditions—including different batch lengths, feed profiles and rates as well as a hunger phase, which was introduced to evaluate the robustness of production–were performed in triplicates, and the results were compared regarding growth behavior, glucose consumption, ethanol and EPG production. During data analysis, multivariate methods were used to significantly reduce the dimensions of the data for visualization, followed by identification of the relevant process drivers during the data/process diagnostics step as well as prediction of the process outcome based on early process information only.

### 3.1. Growth and Carbon Metabolism

The fed-batch phase started after 15 h for cultivations with a lower initial substrate concentration of 20 g L^−1^ and after 16.55 h for cultivations with a higher initial substrate concentration of 30 g L^−1^ as well as for cultivations, where a hunger phase was applied. The initial biomass concentration at t_feedstart_ was 1.70 ± 0.28 g L^−1^.

**Figure 4:**
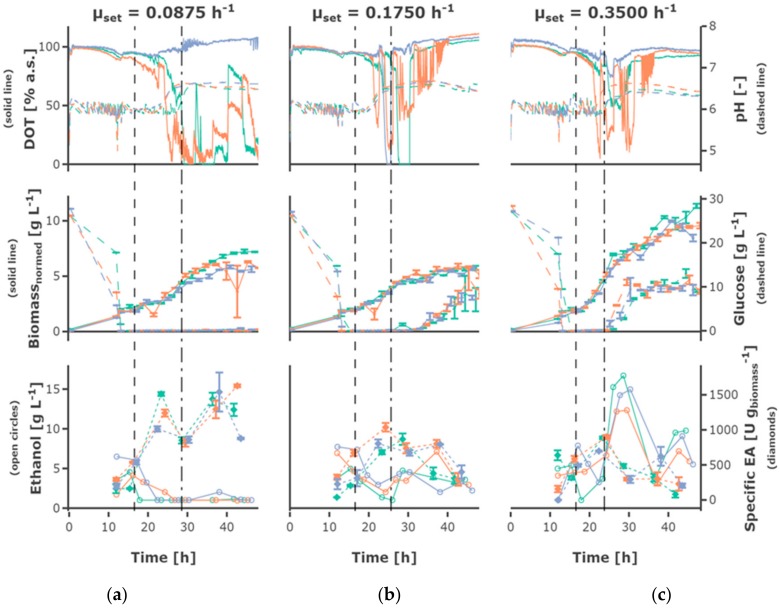
Measurement data is shown regarding the designs 1, 2, and 3—exponential feed with feed rates of (**a**) 0.0875 h^−1^, (**b**) 0.175 h^−1^, and (**c**) 0.35 h^−1^, initial substrate concentration S_0_ = 30 g L^−1^ and no hunger phase. The dissolved oxygen tension (DOT) and pH, biomass, and glucose concentration as well as ethanol concentration and specific enzyme activity are shown. The mean and standard deviation are shown regarding duplicate measurements for biomass and glucose concentration and regarding triplicate measurements for specific enzyme activity. The three MBR cultivations performed under the same conditions are shown in different colors, the start of the feed and the constant fed-batch phase are shown by the vertical dashed, respectively dashed-dotted line. Interactive versions of the plots can be found online [19].

**Figure 5:**
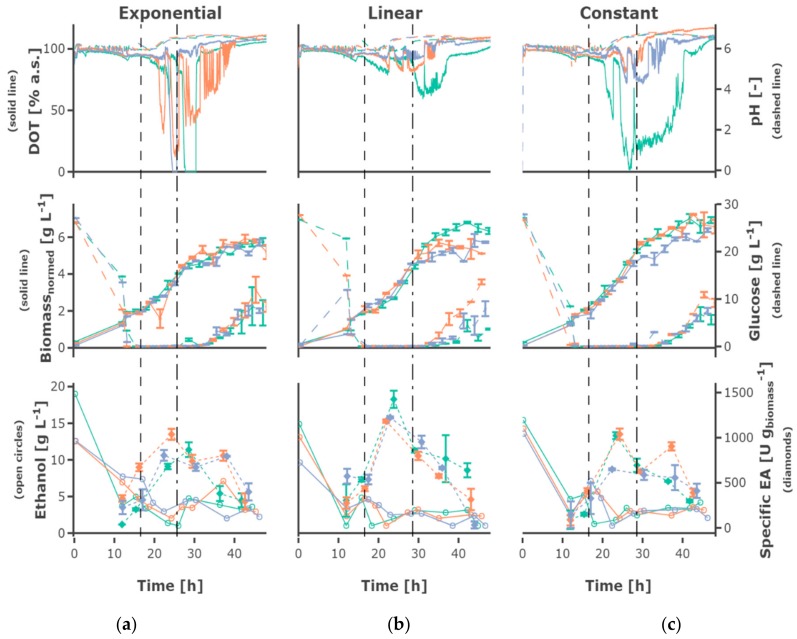
Cultivation data regarding growth at different feed profiles applying a feed rate of 0.175 h^−1^. Measurement data is shown regarding the designs 2, 4 and 9—(**a**) exponential, (**b**) linear and (**c**) constant feed at a feed rate of 0.175 h^−1^ with an initial substrate concentration S_0_ = 30 g L^−1^ and no hunger phase. The DOT and pH, biomass and glucose concentration as well as ethanol concentration and specific enzyme activity are shown. The mean and standard deviation are shown regarding duplicate measurements for biomass and glucose concentration and regarding triplicate measurements for specific enzyme activity. The three MBR cultivations performed under the same conditions are shown in different colors, the start of the feed and the constant fed-batch phase are shown by the vertical dashed, respectively dashed-dotted line. Interactive versions of the plots can be found online [19].

The highest feed rate of 0.35 h^−1^ led to the highest biomass concentrations (Figure 4), the lower feed rates of 0.0875 h^−1^ and 0.175 h^−1^ resulted in comparable growth behavior and final cell density. Regarding exponential growth at different feed rates, similar biomass and glucose concentration were observed for the two different initial substrate concentrations, though a slightly higher biomass concentration was reached for 30 g L^−1^. The feed profile (Figure 5) did not have an observable influence. The cultures grew at a growth rate between of 0.18 ± 0.05 h^−1^ in the batch phase (Figure 5a). During the first fed-batch phase, the growth rate was alternating between µ = 0.04–0.1 h^−1^ for cultivations fed at a feed rate of 0.0875 h^−1^, µ = 0.06–0.1 h^−1^ for a feed rate of 0.175 h^−1^ and µ = 0.15–0.2 h^−1^ for a feed rate of 0.35 h^−1^, respectively (Figure S2). The growth rate was lower at the beginning of the first feed phase—especially for cultivations with hunger phase—indicating a lag phase. After the beginning of the constant fed-batch phase, the growth rate declined. Especially regarding the biomass concentrations, a high reproducibility between the replicates could be reached (Figure S1).

**Figure 6:**
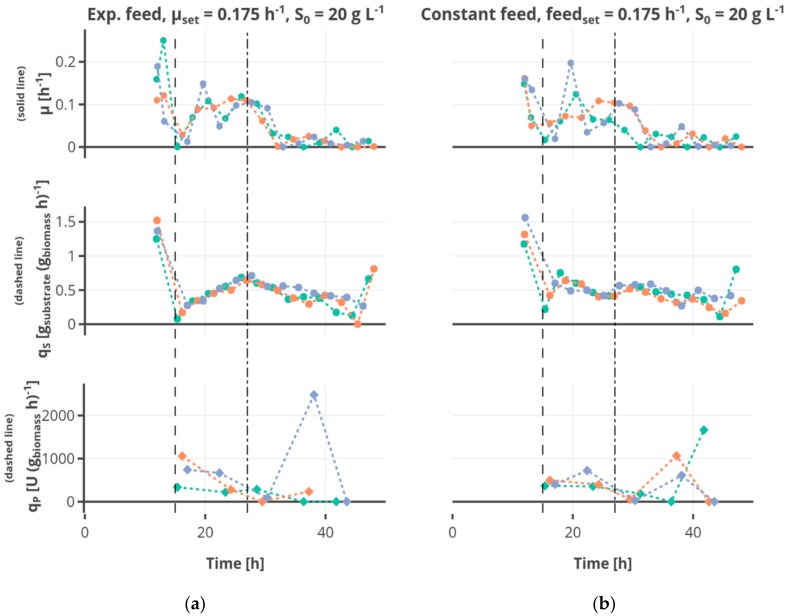
Specific rates for growth rate µ, substrate consumption q_S_ and product formation q_P_ are shown regarding (**a**) exponential feed at a feed rate of 0.175 h^−1^, an initial substrate concentration S_0_ of 20 g L^−1^ and no hunger phase, and regarding (**b**) constant feed at a feed rate of 0.175 h^−1^, an initial substrate concentration S_0_ of 20 g L^−1^ and no hunger phase. The rates are calculated using the mean of the respective measurements. The three MBR cultivations performed under the same conditions are shown in different colors, the start of the feed and the constant fed-batch phase are shown by the vertical dashed, respectively dashed-dotted line. Interactive versions of the plots can be found online [19].

The substrate uptake rate during the batch was 1.34 ± 0.34 g_substrate_ (g_biomass_ h)^−1^. The substrate consumption rate increased in the first feed phase for the cultivations fed exponentially or linearly (see for example Figure 6a). Regarding the cultivations receiving constant feed (see for example Figure 6b), the substrate consumption rate increased in the first part of this feed phase to decrease afterwards due to the decreasing availability of substrate regarding the biomass. After the shift to the constant feed, the substrate consumption rate decreased.

Glucose accumulated depending on the feed rate, for some cultivations fed at the highest feed rate of 0.35 h^−1^ as early as around 20 h cultivation time. Though, glucose accumulation could be observed for all cultivations, even the cultivations fed at the lowest feed rate, after around 40 h of cultivation. After 30–40 h, the cells in all mini-bioreactor cultivations (Figure S1) entered a phase of growth stagnation, although glucose was present in the cultivation medium, partly at high concentrations. 

An increase in pH was observed for all cultivations, starting slightly later in cultivations fed at lower feed rates. The DOT signal was very irregular and oxygen limitation was detected for some cultivations for a short time, possibly due to repeated clogging of the aeration ports for the individual MBRs. However, no effects could be seen on growth and ethanol production regarding cultures with and without short-time oxygen limitation—e.g., regarding the triplicates fed at a growth rate of 0.0875 h^−1^ (Figure 4).

### 3.2. Recombinant Protein Production

The plasmid for EPG production is equipped with the constitutive *ADHI* promoter, so no induction is required. The volumetric and specific enzyme activity increased during the batch and first h of fed-batch phase, though mainly remained constant or decreased towards the end of the fermentation, only increasing slightly for the cultivations fed at a feed rate of 0.0875 h^−1^. Rather constant product formation rates between 150 and 400 U (g_biomass_ h)^−1^ could be observed until 23 h of cultivation. However, the productivity decreased until the end of the cultivation Figure 5c and Figure 6c). The highest final yield and final specific yield for EPG were obtained for the cultivations with an initial substrate concentration of 30 g L^−1^, which were fed exponentially at a µ_set_ = 0.0875 h^−1^ without hunger phase. Specific enzyme activities of up to 1511.9 ± 27.2 U g_biomass_^−1^ were obtained after around 23 h cultivation time (other replicates: 1239.8 ± 49.8 U g_biomass_^−1^; 1013.5 ± 37.1 U g_biomass_^−1^) and 1540.0 ± 278.8 U g_biomass_^−1^ after around 37 h (other replicates: 1439.7 ± 91.3 U g_biomass_^−1^; 1293.8 ± 127.1 U g_biomass_^−1^) (Table S2). The volumetric enzyme activity after 37 h was 9.09 ± 0.58 U mL^−1^ (other replicates: 8.59 ± 1.56 U mL^−1^; 7.82 ± 0.77 U mL^−1^). The cultivations with an initial substrate concentration of 20 g L^−1^ showed a similar EPG expression profile compared to the 30 g L^−1^.

### 3.3. Ethanol Formation

During the batch phase around 5–10 g L^−1^ ethanol are produced. In cultivations fed at a rate of 0.0875 h^−1^ the remaining ethanol was taken up by co-metabolism of glucose and ethanol, and decreased below the detection rate. Ethanol was present at rather constant concentrations of around 3 g L^−1^ in cultivations fed at a rate of 0.175 h^−1^, while ethanol accumulating up to 15 g L^−1^ occurs in cultivations fed at a rate of 0.35 h^−1^. In most cultivations fed at the rate of 0.35 h^−1^ the ethanol concentration declined after a cultivation time of 30 h to increase again until the end of the cultivation.

### 3.4. Multivariate Analysis for Information Extraction

**Figure 7:**
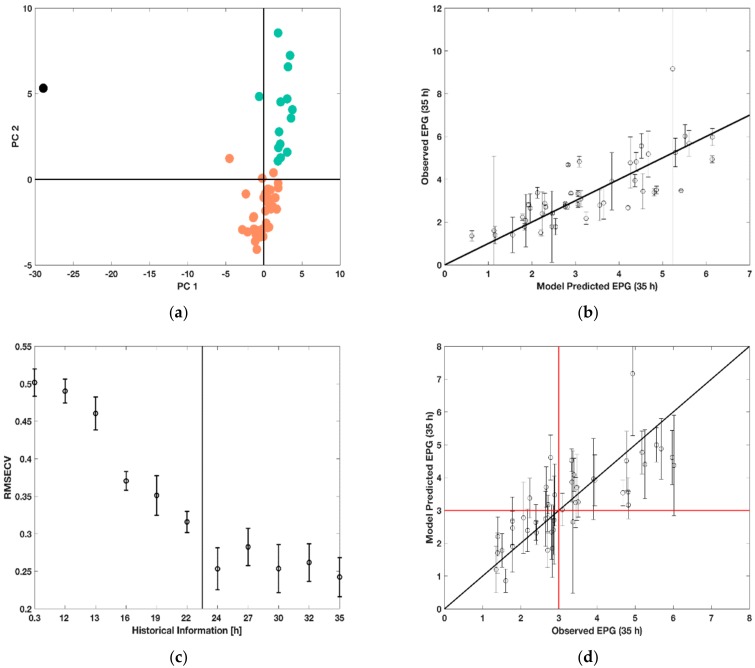
Real-time outlier identification and batch characterization using principal component analysis (PCA). (**a**) Clusters 1 (red circles) with 17 observations, cluster 2 (green circles) with 30 observations and the outlier (black circle) detected after 24 h of cultivation; (**b**) Prediction of a partial least square regression (PLSR) model using all history and variable selection against the experimental values of volumetric EPG activity at 35 h. (**c**) Root mean square error of cross validation (RMSECV) (see Section 2.10) of the PLSR models built based on different amounts of history (with variable selection). (**d**) Experimental values of volumetric EPG activity at 35 h of batch against the model prediction built using the history until 24 h (with variable selection).

First, BWU–PCA was performed for each measurement time point by using the historic process information until the considered time point for analysis. Thus, at every time point, the routine was able to identify batches showing abnormal behavior. Figure 7 shows score plots of the BWU-PCA scores for all 48 runs incorporating their process history until 22 h. It can be identified that one run falls distinctly apart from all the other batches at both time points, i.e., that such abnormality can be detected early in the process. This was identified to be run 46 (marked in black in Figure 7a), which experienced a failure during the experiments. The pH and DOT sensors were not working properly resulting in incorrect culture handling. For future analysis, this outlier was removed. 

In addition, runs that were similar were identified using the k-means clustering algorithm resulting in three clusters as shown in Figure 7a in orange, green and black. A decision tree analysis highlighted that the two characteristic clusters (marked orange and green) were determined by the feeding rate and were segregated into the lower feed rates of 0.0875 h^−1^ and 0.175 h^−1^ and the high feed rate of 0.35 h^−1^. However, the other manipulated variables in the experimental design, i.e., feeding profile, hunger phase and initial substrate concentration did not show significance in determining the similarity of runs. Nonetheless, these might be indeed important to further understand peculiarities of the process behavior in each of the major clusters as well as to explain the product characteristics. It is important to highlight that the abnormal behavior of run 46 can be detected within the first hour of the process duration, while the distinctly different evolution of the process can be segregated from 22 h. Although in this simple case the outlier can be detected visually based on the process information, such a tool is generally useful to identify pro-actively abnormal and different process behavior so to suggest on improved operating conditions or abort the process.

With regards to the second goal, historic PLSR models were built to predict the analytically costly EPG activity based on the simple-to-access process measurements. For the EPG prediction at two measurement times, 22 h and 35 h, an average RMSECV of 21.45 ± 12% and 23.30 ± 12% was obtained, respectively. The prediction results for the latter case are additionally visualized in Figure 7b where one can observe that most of the 47 runs are decently predicted while few runs are either under- or over-estimated. The limitation of such predictions can be noise in the measurements of process variables and EPG (highlighted by error bars). Especially, the analytics for the latter should be improved to decrease variability and provide a more consistent basis for prediction. On the other hand, the linear structure of the model is likely not to capture all the peculiarities of the biological system, so that mechanistically valid non-linear terms are likely to be of advantage.

A further goal of this predictive analysis was the evaluation of the possibility to forecast the final volumetric EPG activity based on a shorter duration of process history, i.e., not only to build a soft sensor for the protein activity based on easier-to-access process measurements but also to anticipate the activity in advance so to provide an early basis for decision taking. Figure 7c shows the RMSECV distribution obtained for different amounts of process history used for prediction. One can observe that the process outcome can already be accurately predicted after 24 h. The two significant drops of the RMSECV distributions at 15 and 21 h signify that important information on the process characteristics are added here. The latter observation is also in line with the one from Figure 7a where only after 22 h a clear separation of the two process regimes was evident. The first observation is very likely to be related to the start of feeding, while the second observation could be interpreted as the time point when the response of the fermentation process to the culture conditions including the feeding profile is clearly established. The importance of such analysis is highlighted in Figure 7d, which shows the PLSR model predictions based on process history until 24 h to forecast the final volumetric EPG activity. Error bars signify the standard deviation of the predictions, while the red cross helps to distinguish low productivity runs (EPG < 3 U mL^−1^) from high productivity runs (EPG > 3 U mL^−1^). Although, the error is rather high regarding the model prediction, probably due to deviations between the triplicates of cultivations performed under the same conditions and measurement errors, the model can detect a trend in the culture behavior. With few exceptions, after two thirds of the process duration the model can therefore clearly forecast whether under the given operating conditions, the volumetric EPG activity 11 h later will be low or high. This enables in the future to optimize the process conditions in real-time based on such predictions. However, further improvements of the model could be achieved by increasing the accuracy of the measurements and improving the outlier detection.

Besides the consideration until which time point the process must be quantified, Table 2 targets the analysis based on the variable selection routine, which time points in particular provide important and unique process information to predict volumetric EPG activity. In fact, the sampling scheme could be drastically reduced, measuring instead of 28 samples (11 for biomass, 11 for glucose and 6 for ethanol) only 17 samples, while retaining the predictive power of the corresponding two models.

Finally, the multi-target characteristics of yeast fed-batch development shall be considered, namely the adaptation of the process operation model so to increase the product formation while minimizing the ethanol production [16]. This was addressed through regression tree analysis of the manipulated process conditions to the four characteristics of interest, i.e., the volumetric EPG activity and ethanol concentration, both measured at 22 h and 35 h. Table 3 shows optimal paths for each of the four characteristics resulting in similar distributions of the considered variable (represented by mean and standard deviation). For instance, for ethanol at 22 h three possible process operation selections were identified, while for the volumetric EPG activity at 35 h one sequence of critical decisions for process variables was obtained. Regarding all four characteristics, recurring conditions were exponential feeding at a feeding rate of 0.0875 h^−1^ with an initial substrate concentration of 30 g L^−1^ and no hunger phase, which were thus identified as the most appropriate conditions. The aim was not to identify the optimal process conditions based on the single highest yield but to find those leading to an optimal process outcome in most cases. The regression tree analysis confirms the observations made for ethanol accumulation and volumetric EPG activity in the previous sections but additionally identifies other equivalent possibilities. With further targets to be considered in the future as well as additional process parameters tested, this approach offers a stream-lined procedure for model-based decision taking for process optimization.

## 4. Discussion

In this study, 16 experimental conditions were carried out in triplicates in 48 MBRs to evaluate the influence of substrate availability and feeding strategy on recombinant protein production in *S. cerevisiae*. Cultivation, sampling and at-line analysis were performed automatically on a HT platform. Data handling—from raw data processing to the visualization and predictive modeling—was performed standardized and automated with minimal human input to rapidly gain process information and decision support from the enormous amount of data, which was collected during the experiment. 

MBR platforms allow to combine the advantages of both microtiter plates and benchtop-scale bioreactors resulting in high experimental throughput and high information gain [4]. They thus are an important step towards consistent bioprocess development. Multiple replicates of the cultivations with the same experimental conditions were included and high comparability between experiments was achieved as the batch variability was reduced compared to the sequential approach. The latter is also important, as it has been shown that the history of the cells has a strong influence on the cultivation [16,47].

A maximum specific product formation rate of 400 U (g_biomass_ h)^−1^ was observed at the lowest feed rate of 0.0875 h^−1^ and 250 U (g_biomass_ h)^−1^ at a feed rate of 0.175 h^−1^. This is comparable to the rates achieved at similar dilution rates of 0.08 to 0.11 h^−1^ in change-stat cultivations of *S. cerevisiae* AH22 expressing EPG as reported earlier [16]. The actual growth rate was around 30% of the set growth rate and was thus less than expected. Reasons for the growth inhibition might be the metabolic burden of recombinant protein production [48], the negative effect of ethanol on sugar and amino acid transport [49] or—in case of the higher feed rates—of the overflow metabolism [50]. Also, oscillations in substrate availability—which are introduced here by the semi-continuous feed—have been shown to lead to a reduction in growth [51,52]. The growth arrest, occurring here after 30–40 h, could be caused by the metabolic stresses which are applied during recombinant protein production [53], the depletion of some medium components or the accumulation of self-produced toxic by-products, including but not exclusively ethanol [35]. It was shown that accumulation of lactic and acetic acid lead to reduced growth and substrate consumption and increase ethanol production [54]. However, during the cultivations described here, neither lactic nor acetic acid were accumulated to concentrations reported to have a negative influence (Table S3). Ethanol production, reducing the yield, started in the cultivations fed at feed rate of 0.175 h^−1^, which is lower than the critical dilution rate for ethanol production µ_crit_ = 0.2 h^−1^ in change-stat cultivations [16]. Again, oscillating sugar concentration might be the reason for increased ethanol production [51]. While the intermittent feeding in the MBR system is able to resemble substrate-gradients in large-scale production processes, and thus is a suitable tool for scale-down simulation [41], it results in cell stress. An improvement of the cultivation conditions can be achieved by enzymatic glucose release mimicking continuous fed-batch conditions [55].

Principal component analysis supported as a visualization tool the identification of outliers and varying process behavior. In the future, such tools can be integrated into the experiments to stop strongly deviating runs or identify potentially abnormal features to adapt the corresponding process conditions and control them towards the targets. Predictive models based on PLSR showed that soft sensors can be built based on simple-to-access process data to reliably quantify EPG activity. Such predictions can be obtained only after two thirds of the process duration enabling to forecast the productivity of given operation conditions and support decision taking to improve or abort the low producing runs. Moreover, the embedded variable selection tool enabled to quantify the minimal number and characteristic time points of measurement yielding sufficient information content, i.e., an effective process analytical scheme. Given the two targets, namely high volumetric yield and low ethanol production, super-imposed regression trees enabled to identify important process parameters and their desired levels to fulfill these targets. A combination of the lowest feed rate of 0.0875 h^−1^, an exponential feed with an initial substrate concentration of 30 g L^−1^ and no hunger phase resulted in the highest volumetric yield of ~8.5 U mL^−1^ (mean of triplicates) in comparison to a volumetric yield of ~2.1 U mL^−1^ regarding the design with the lowest yield. For future analysis, these tools must be directly implemented into the experimental platform so to enable real-time decision taking and process optimization. Like this, an even better evaluation of the potential of such automated HT technology for efficient process development can be achieved. The real-time adaptations based on the model predictions will pave the path to better process understanding and the creation of a digital twin of the process. Several sequential iterations of such an experiment will enable to not only design an optimal process in the Quality by Design (QbD) perspective, i.e., to identify the settings of the Critical Process Parameters (CPP) resulting in optimal Critical Quality Attributes (CQA), but will also provide a technologically and economically optimal operation procedure with regards to the dynamic control structure, sampling scheme and reporting base for the involved decision takers.

Bottlenecks of HT methods still exist but have shifted from experimental throughput to offline analysis, data handling and evaluation [4]. Frequent sampling of up to 48 cultivations in parallel over several days results in many samples, i.e., during the presented cultivation 744 samples were taken. Although methods and devices for HT sample preparation and analysis already exist—e.g., a HT method for cell disruption [13]—exploiting the full potential of HT screening remains a challenge, particularly with regards to online product quality characteristics quantification [56], and requires fast analytical methods suitable for parallelization. Thus, quantitative tools for prediction and decision support will remain a key enabler of the successful realization of such automated technology. In this work, a PLSR based model was developed to predict the EPG activity based on simple-to-access process quantities. More dynamic measurements of this quantity would in the future enable to build a real-time sensor for this product characteristic. Moreover, the developed models can be further intensified through integration of existing process knowledge to generate hybrid process models [57].

The developed method provides a remarkable advancement towards the goals of industry 4.0 based on an efficient, parallelized and automated system for cultivation and analytics as well as a predictive digital framework for data management and analysis. A further intensification of the technology towards additional analytical capabilities, complete integration of hardware and software technologies, enabling adaptive process control and integration of all involved stakeholders and process know-how into such a self-learning digital platform, will revolutionize the current procedures of process development through a broadly applicable, automated robotic platform.

## Figures and Tables

**Table 1:** Cultivation details. The experimental conditions regarding each mini-bioreactor are shown, including the fed-batch profile, feed rate, initial substrate concentration S_0_ and occurrence of a hunger phase.

Condition	Profile	Feed Rate (h^−1^)	S_0_ (g L^−1^)	Hunger Phase (h)
1	Exponential	0.0875	30	-
2	Exponential	0.175	30	-
3	Exponential	0.35	30	-
4	Constant	0.175	30	-
5	Exponential	0.0875	20	-
6	Exponential	0.175	20	-
7	Exponential	0.35	20	-
8	Constant	0.175	20	-
9	Linear	0.175	30	-
10	Linear	0.35	30	-
11	Linear	0.175	30	2
12	Linear	0.35	30	2
13	Linear	0.175	20	-
14	Linear	0.35	20	-
15	Exponential	0.0875	20	
16	Exponential	0.35	20	2

**Table 2:** Important measurement times for biomass, glucose and ethanol concentration that provide crucial information for the volumetric endopolygalacturonase (EPG) activity prediction model.

	Biomass (h)	Glucose (h)	Ethanol (h)
EPG (22 h)	12, 13, 16, 19, 22	0.3, 16, 19	16, 22
EPG (35 h)	16, 22, 27, 32	12, 13, 27, 30	16, 22, 35

**Table 3:** Optimal conditions regarding the feed rate and profile, the initial substrate concentration S_0_ and the presence of a hunger phase identified by decision tree analysis for high volumetric EPG activity and low ethanol production. Each row in the table corresponds to an optimal combination of process conditions for the considered target variable. The total number of runs performed under these conditions as well as the mean and standard deviation of the ethanol concentration and volumetric EPG activity is given regarding those runs. The symbol ‘-’ indicates that any value of this variable is acceptable. Conditions which lead to an optimal result regarding both ethanol concentration and volumetric EPG activity at both time points are underlined.

	Feed Rate (h^−1^)	Feed Profile	S_0_ (g L^−1^)	Hunger Phase (h)	Number of Runs (-)	Mean	Std
Ethanol (22 h) (g L^−1^)	0.0875 0.175 0.175	- Linear Constant/Exponential	- - 30	- - -	9 9 6	1299 1521 1498	418 577 518
Ethanol (35 h) (g L^−1^)	0.0875 0.175	Linear/Exponential Linear	- -	0 -	6 6	951 1381	350 568
EPG (22 h) (U mL^−1^)	- 0.175	Constant/Exponential Linear	30 30	- 0	12 6	2.6 2.4	0.7 0.8
EPG (35 h) (U mL^−1^)	0.0875	Constant/Exponential	-	-	6	5	2

## Supplementary Materials

The following are available online at http://www.mdpi.com/2306-5354/5/4/101/s1, Figure S1: Cultivation data regarding growth at different applied cultivation conditions, Figure S2: Specific rates for growth µ, substrate consumption q_S_ and product formation q_P_ regarding different applied cultivation conditions, Table S1: Layout of experimental conditions, Table S2: Volumetric and specific enzyme activity (EA) after 23 h and 37 h cultivation time, Table S3: Acetate, lactate, ammonia and phosphate concentration at 32 h cultivation time.

## Appendix A

The linear feed *F_lin_* (L h^−1^) was calculated by:

(A1) $$F_{lin}\left(t\right)=F_{0}+a\cdot t$$

where *a* represents the linear increase in the feed rate. The slope *a* is determined by the integral of the exponential feed *A* as follows:

(A2) $$A=\int_{t_{0}}^{t_{end}}\left(F_{0}+a\cdot t\right)dt={\left[F_{0}\cdot t+\frac{1}{2}a\cdot t^{2}\right]}_{t_{0}}^{t_{end}}=F_{0}\cdot \left(t_{end}-t_{0}\right)+\frac{1}{2}a\cdot \left(t_{end}^{2}-t_{0}^{2}\right)\;↔\;a=\frac{2\left(A-F_{0}\cdot \left(t_{end}-t_{0}\right)\right)}{t_{end}^{2}-t_{0}^{2}}$$

The constant feed *F_const_* (L h^−1^) is calculated as follows:

(A3) $$F_{const}\left(t\right)=b=\frac{A}{t_{end}-t_{0}}$$
