# Supplementary material for: Accelerated Bioprocess Development of Endopolygalacturonase-Production with Saccharomyces cerevisiae Using Multivariate Prediction in a 48 Mini-Bioreactor Automated Platform
Source: Bioengineering (Basel). 2018 Nov 21;5(4):101. doi: 10.3390/bioengineering5040101 (PMC6316240; doi:10.3390/bioengineering5040101)
Supplement: Supplementary file 1 [file bioengineering-05-00101-s001.pdf]

bioengineering

Supplementary information

Figure S1 and S2 show the cultivation data and specific rates for growth μ, substrate consumption q_S and product formation q_P for all mini-bioreactors. Mini-bioreactor 46 is not included as it was identified as outlier.

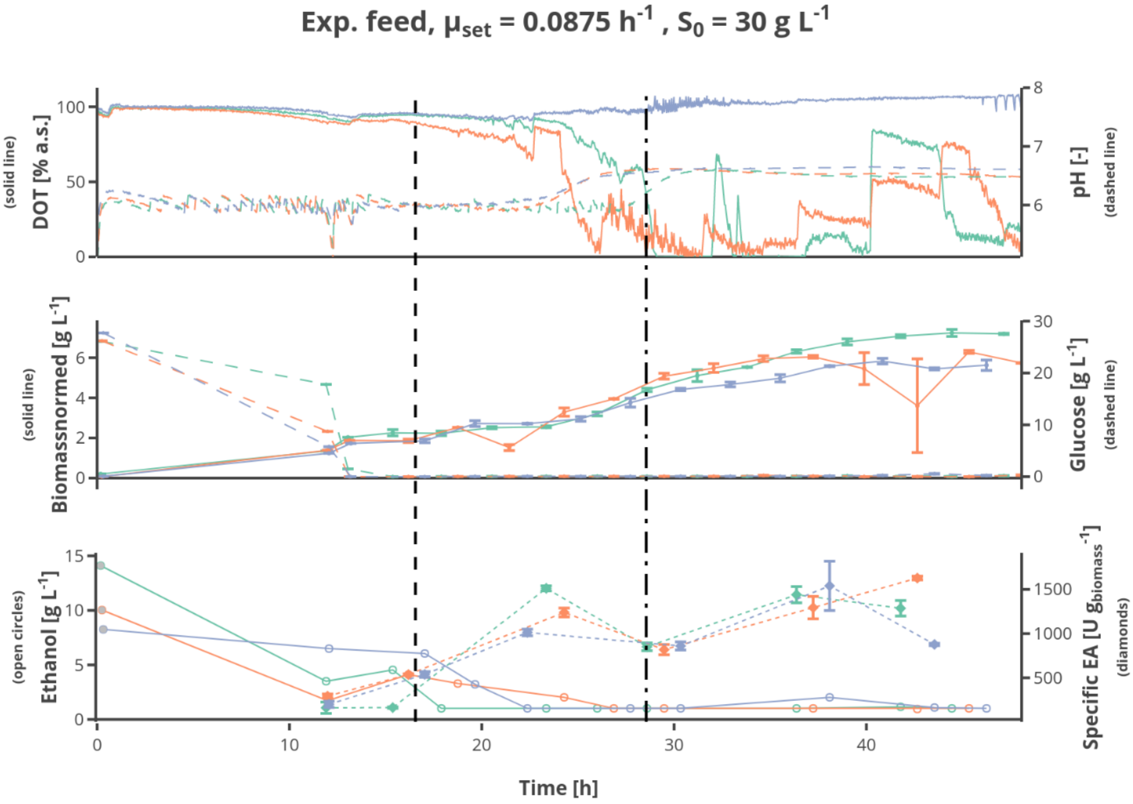

(**a**)

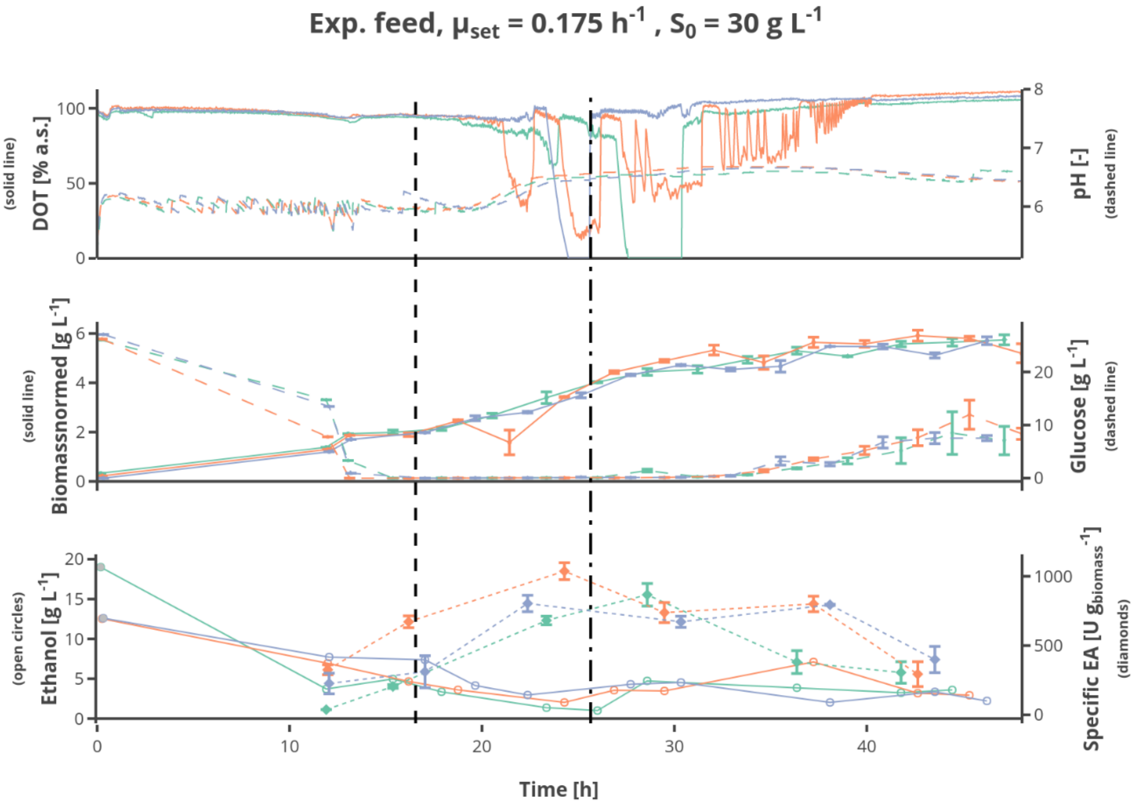

Exp. feed, μ_{set} = 0.175 h^{-1} , S_0 = 30 g L^{-1}
(solid line)
DOT [% a.s.]
pH [-]
(dashed line)
(solid line)
Biomassnormed [g L^{-1}]
Glucose [g L^{-1}]
(dashed line)
(open circles)
Ethanol [g L^{-1}]
Specific EA [U $g_{biomass}^{-1}$]
(diamonds)
Time [h]

(**b**)

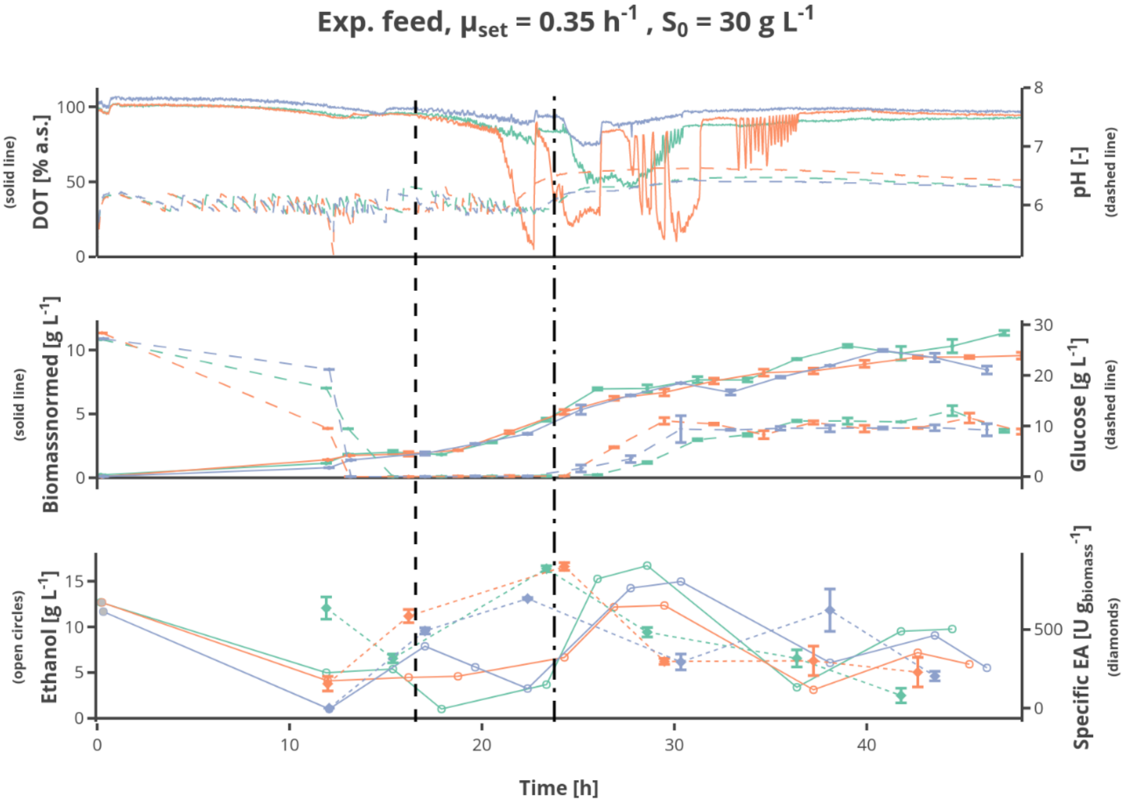

Exp. feed, μ_{set} = 0.35 h^{-1} , S_0 = 30 g L^{-1}
(solid line)
DOT [% a.s.]
pH [-]
(dashed line)
(solid line)
Biomassnormed [g L^{-1}]
Glucose [g L^{-1}]
(dashed line)
(open circles)
Ethanol [g L^{-1}]
Specific EA [U $g_{biomass}^{-1}$]
(diamonds)
Time [h]

(**c**)

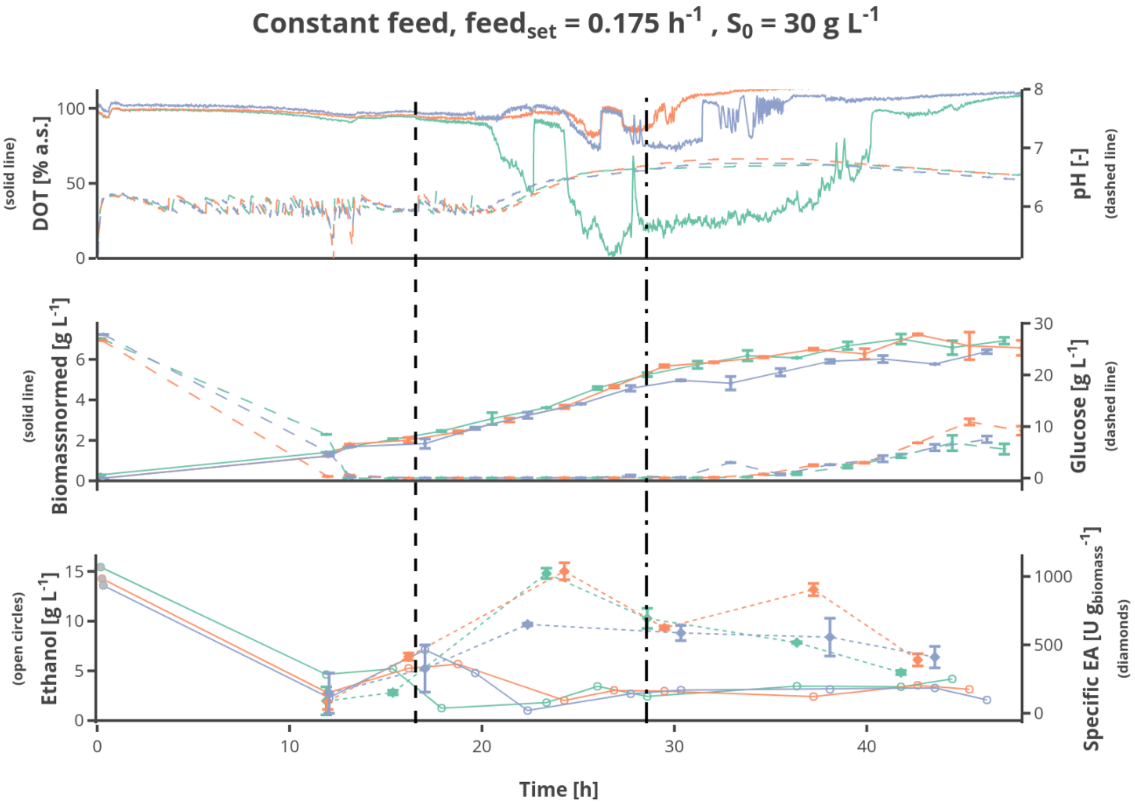
Constant feed, feed$_{set}$ = 0.175 h^{-1}, S$_0$ = 30 g L^{-1}
(solid line)
DOT [% a.s.]
pH [-]
(dashed line)
(solid line)
Biomassnormed [g L^{-1}]
Glucose [g L^{-1}]
(dashed line)
(open circles)
Ethanol [g L^{-1}]
Specific EA [U g$_{biomass}$$^{-1}$]
(diamonds)
Time [h]

(**d**)

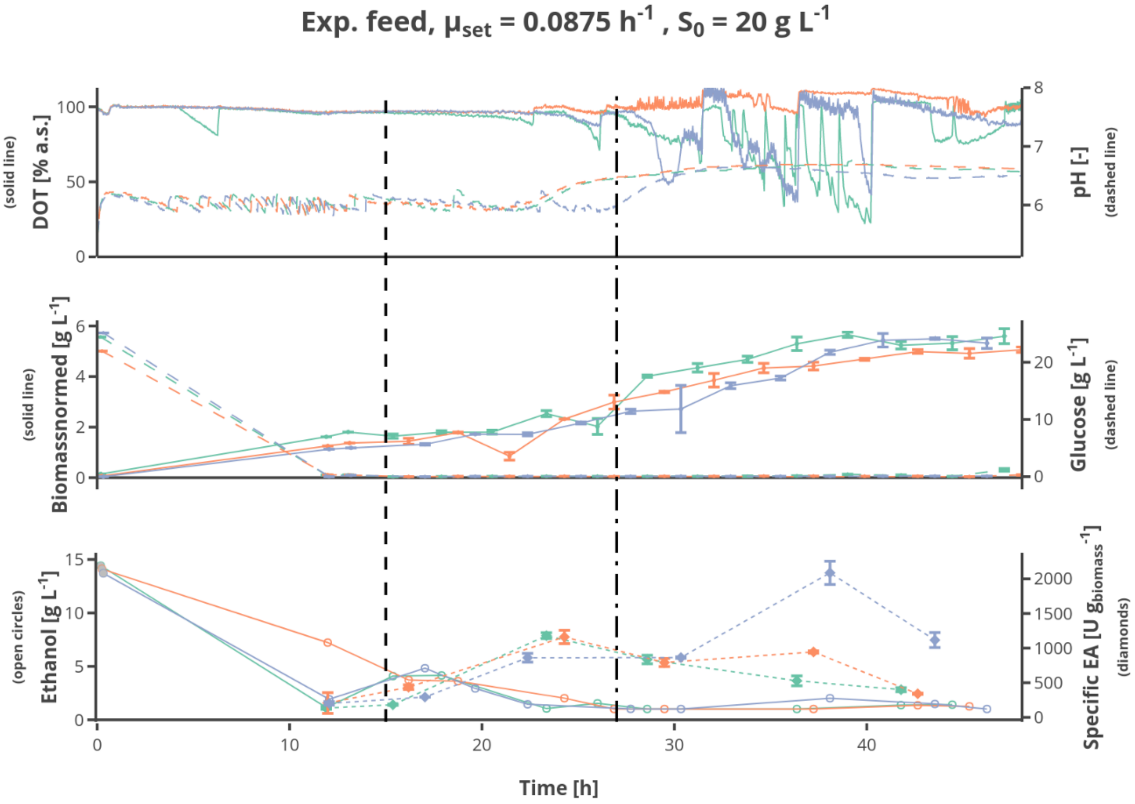
Exp. feed, μ$_{set}$ = 0.0875 h^{-1}, S$_0$ = 20 g L^{-1}
(solid line)
DOT [% a.s.]
pH [-]
(dashed line)
(solid line)
Biomassnormed [g L^{-1}]
Glucose [g L^{-1}]
(dashed line)
(open circles)
Ethanol [g L^{-1}]
Specific EA [U g$_{biomass}$$^{-1}$]
(diamonds)
Time [h]

(**e**)

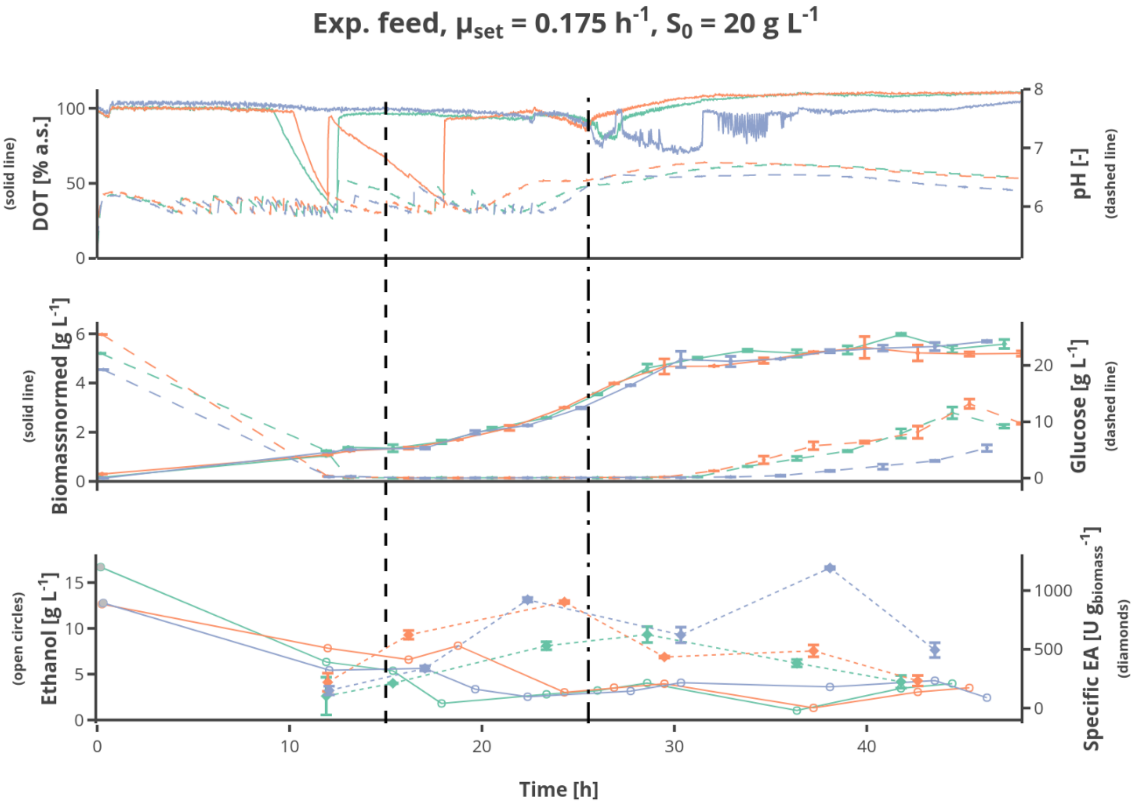
Exp. feed, μset = 0.175 h-1, S0 = 20 g L-1
(solid line)
DOT [% a.s.]
pH [-]
(dashed line)
(solid line)
Biomassnormed [g L-1]
Glucose [g L-1]
(dashed line)
(open circles)
Ethanol [g L-1]
Specific EA [U gbiomass-1]
(diamonds)
Time [h]

(**f**)

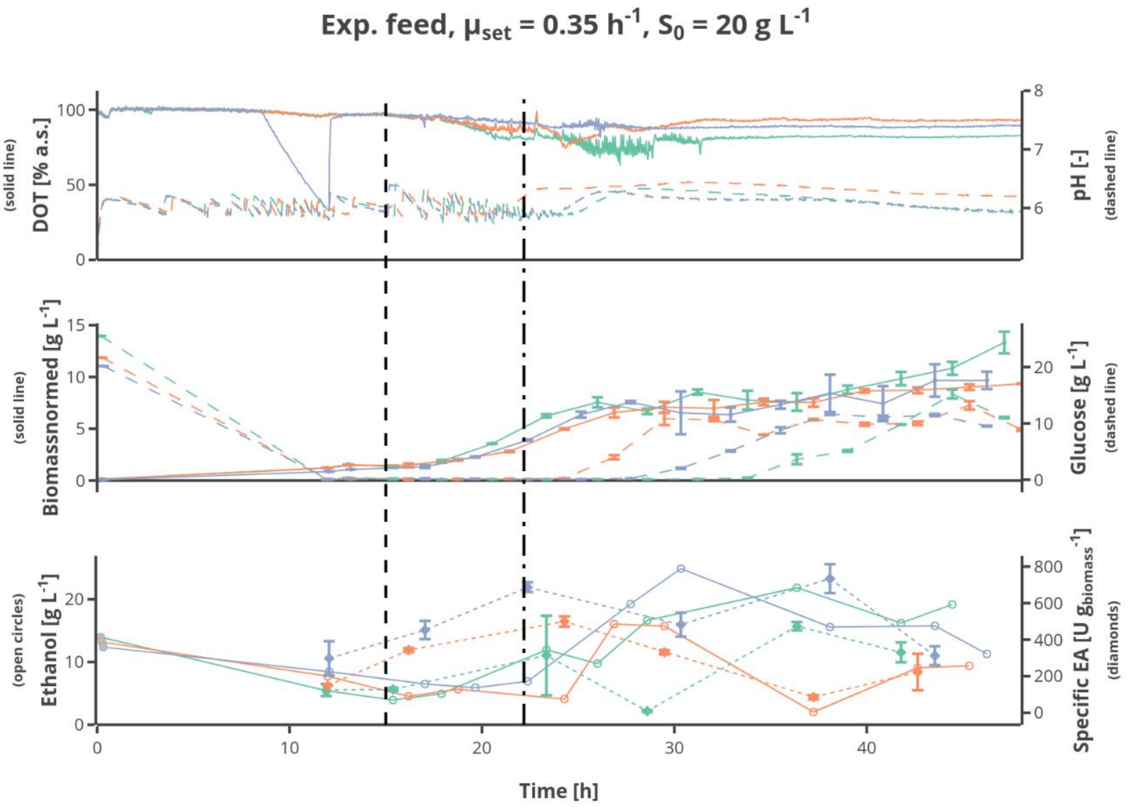
Exp. feed, μset = 0.35 h-1, S0 = 20 g L-1
(solid line)
DOT [% a.s.]
pH [-]
(dashed line)
(solid line)
Biomassnormed [g L-1]
Glucose [g L-1]
(dashed line)
(open circles)
Ethanol [g L-1]
Specific EA [U gbiomass-1]
(diamonds)
Time [h]

(**g**)

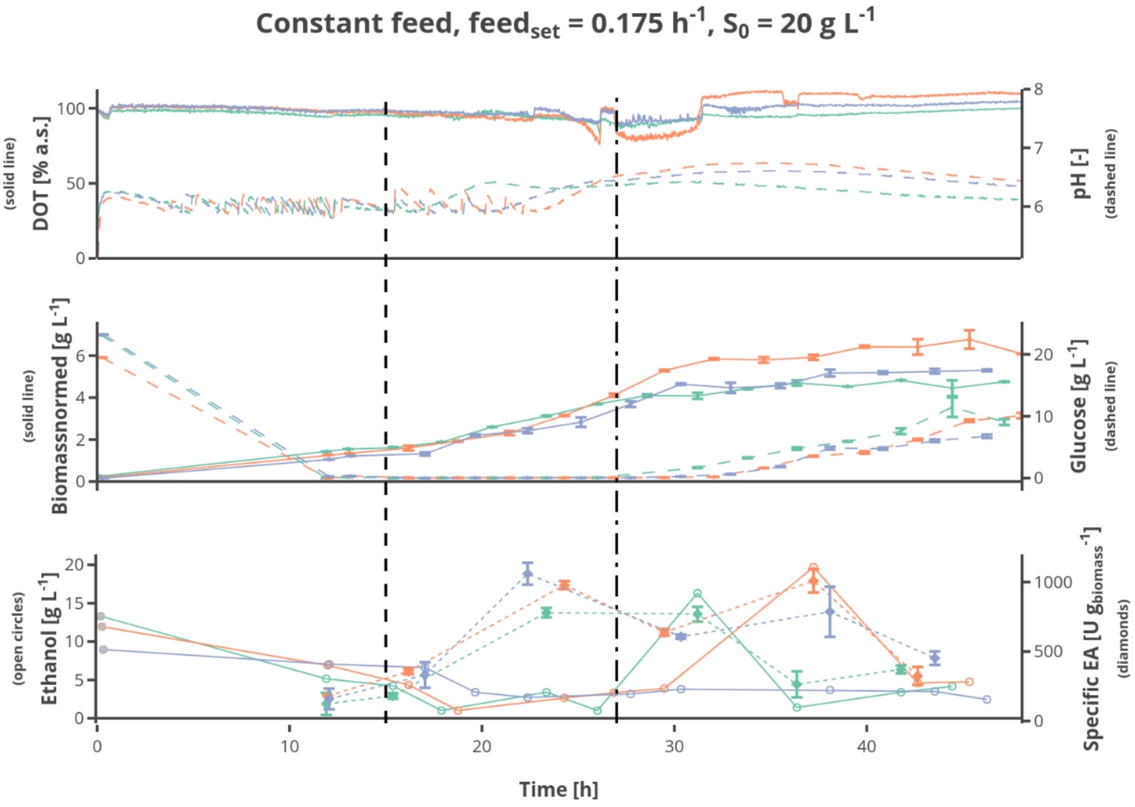
Constant feed, feed$_{set}$ = 0.175 h^{-1}, S$_0$ = 20 g L^{-1}
DOT [% a.s.]
(solid line)
pH [-]
(dashed line)
Biomassnormed [g L^{-1}]
(solid line)
Glucose [g L^{-1}]
(dashed line)
Ethanol [g L^{-1}]
(open circles)
Specific EA [U g$_{biomass}$$^{-1}$]
(diamonds)
Time [h]

(**h**)

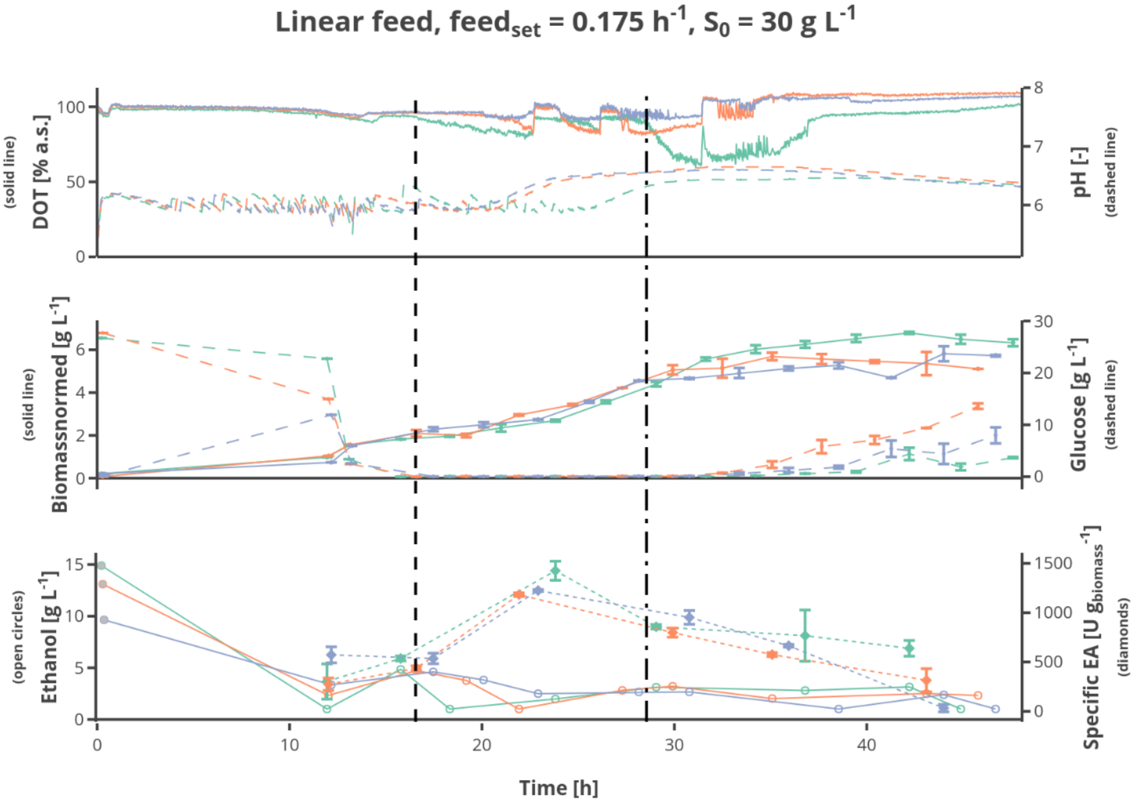
Linear feed, feed$_{set}$ = 0.175 h^{-1}, S$_0$ = 30 g L^{-1}
DOT [% a.s.]
(solid line)
pH [-]
(dashed line)
Biomassnormed [g L^{-1}]
(solid line)
Glucose [g L^{-1}]
(dashed line)
Ethanol [g L^{-1}]
(open circles)
Specific EA [U g$_{biomass}$$^{-1}$]
(diamonds)
Time [h]

(**i**)

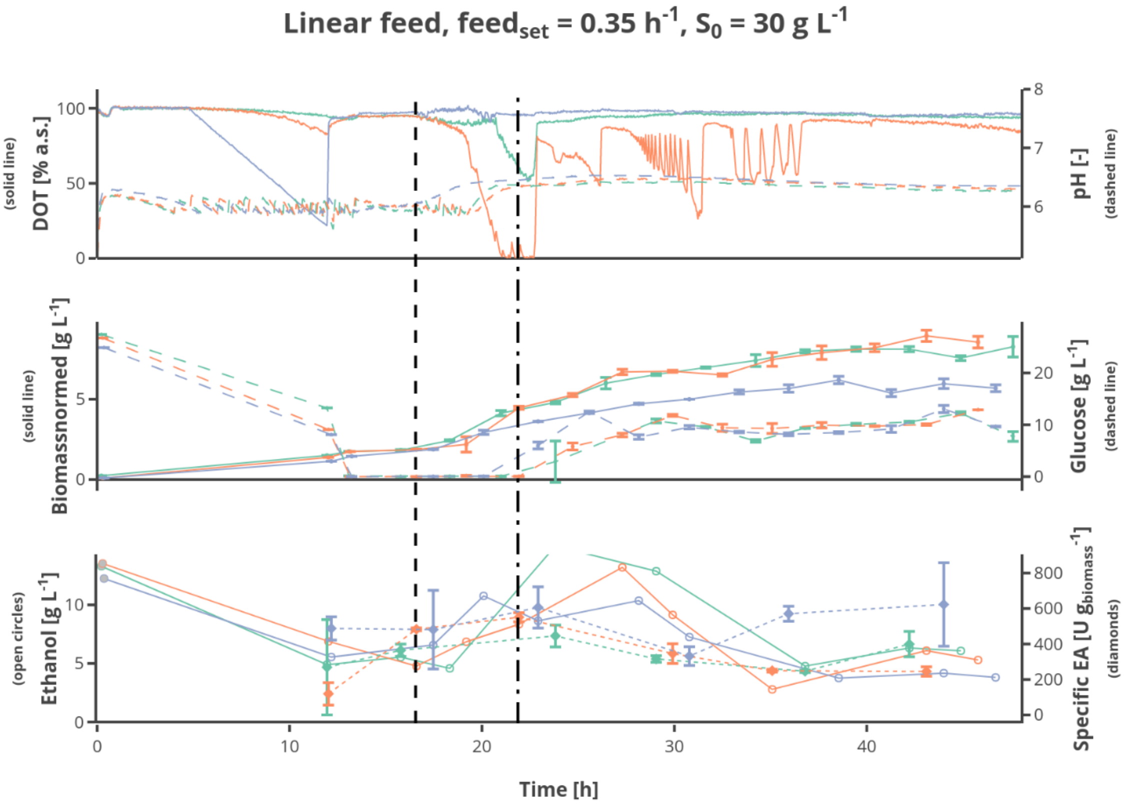
Linear feed, feedset = 0.35 h-1, S0 = 30 g L-1
DOT [% a.s.]
(solid line)
pH [-]
(dashed line)
Biomassnormed [g L-1]
(solid line)
Glucose [g L-1]
(dashed line)
Ethanol [g L-1]
(open circles)
Specific EA [U gbiomass-1]
(diamonds)
Time [h]

(j)

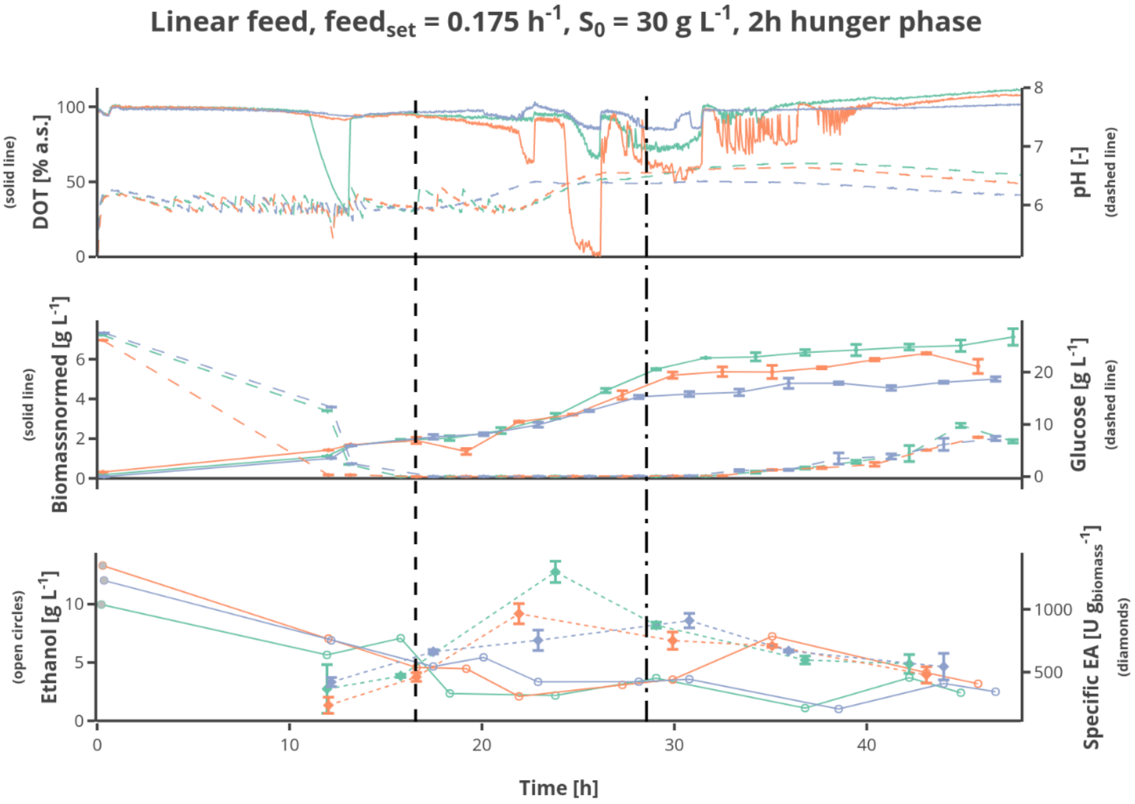
Linear feed, feedset = 0.175 h-1, S0 = 30 g L-1, 2h hunger phase
DOT [% a.s.]
(solid line)
pH [-]
(dashed line)
Biomassnormed [g L-1]
(solid line)
Glucose [g L-1]
(dashed line)
Ethanol [g L-1]
(open circles)
Specific EA [U gbiomass-1]
(diamonds)
Time [h]

(k)

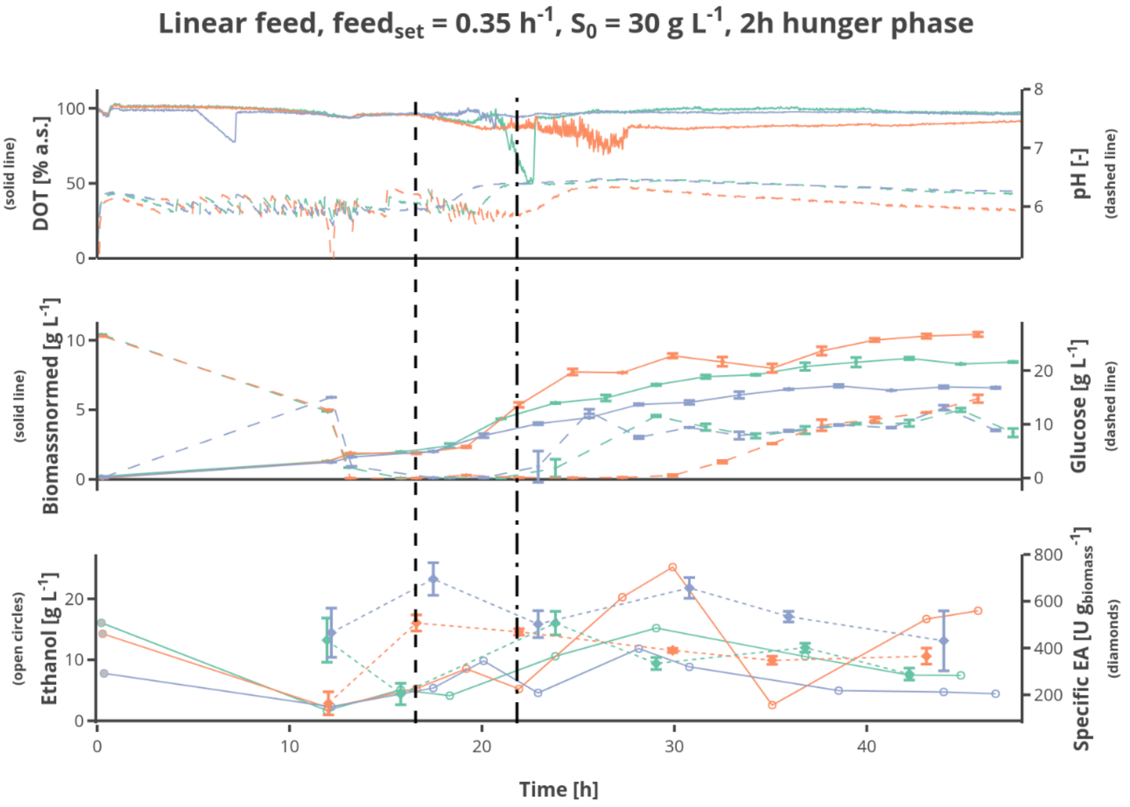
Linear feed, feed$_{set}$ = 0.35 h^{-1}, S$_0$ = 30 g L^{-1}, 2h hunger phase
(solid line)
DOT [% a.s.]
pH [-]
(dashed line)
(solid line)
Biomassnormed [g L^{-1}]
Glucose [g L^{-1}]
(dashed line)
(open circles)
Ethanol [g L^{-1}]
Specific EA [U g$_{biomass}^{-1}$]
(diamonds)
Time [h]

(**l**)

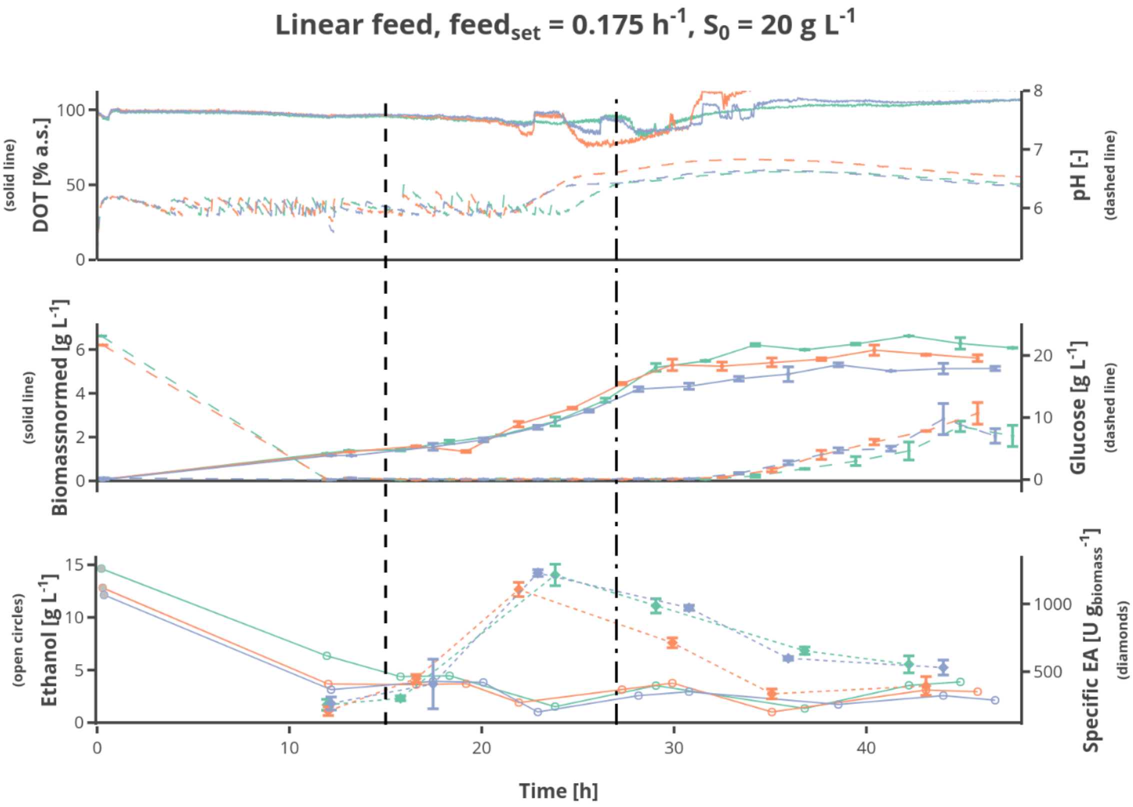
Linear feed, feed$_{set}$ = 0.175 h^{-1}, S$_0$ = 20 g L^{-1}
(solid line)
DOT [% a.s.]
pH [-]
(dashed line)
(solid line)
Biomassnormed [g L^{-1}]
Glucose [g L^{-1}]
(dashed line)
(open circles)
Ethanol [g L^{-1}]
Specific EA [U g$_{biomass}^{-1}$]
(diamonds)
Time [h]

(**m**)

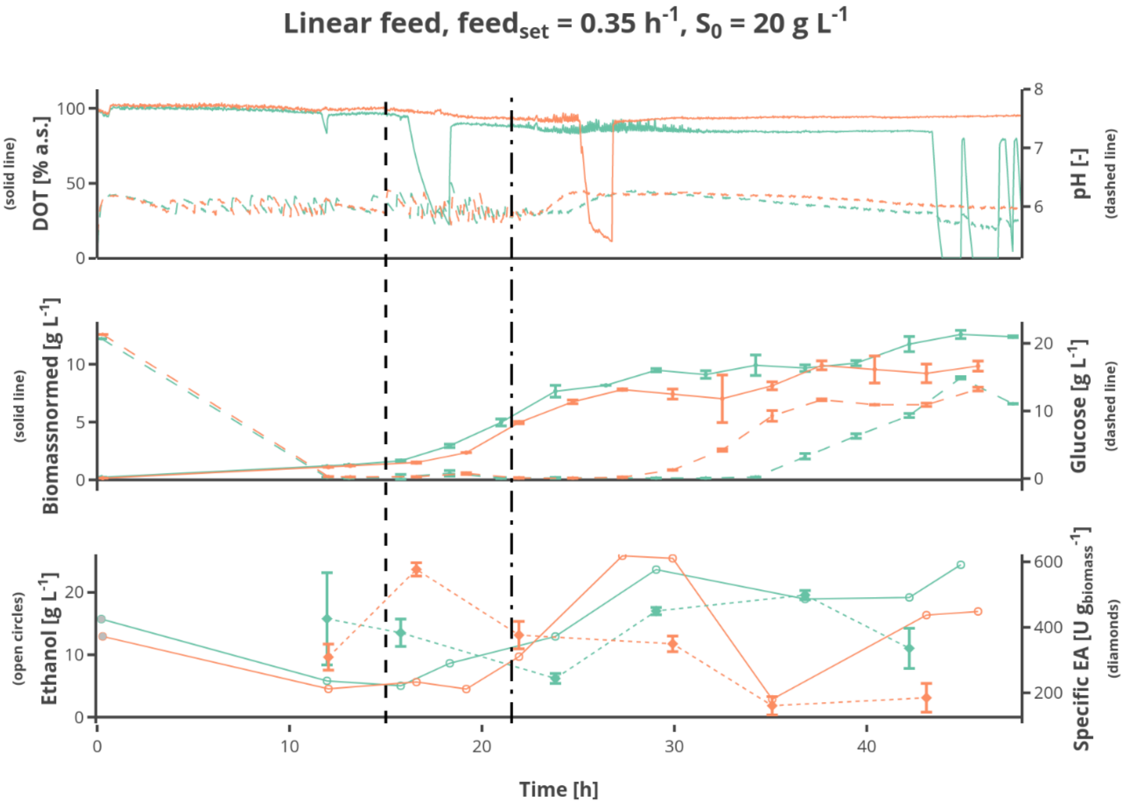
Linear feed, feedset = 0.35 h-1, S0 = 20 g L-1
(solid line)
DOT [% a.s.]
pH [-]
(dashed line)
(solid line)
Biomassnormed [g L-1]
Glucose [g L-1]
(dashed line)
(open circles)
Ethanol [g L-1]
Specific EA [U gbiomass-1]
(diamonds)
Time [h]

(**n**)

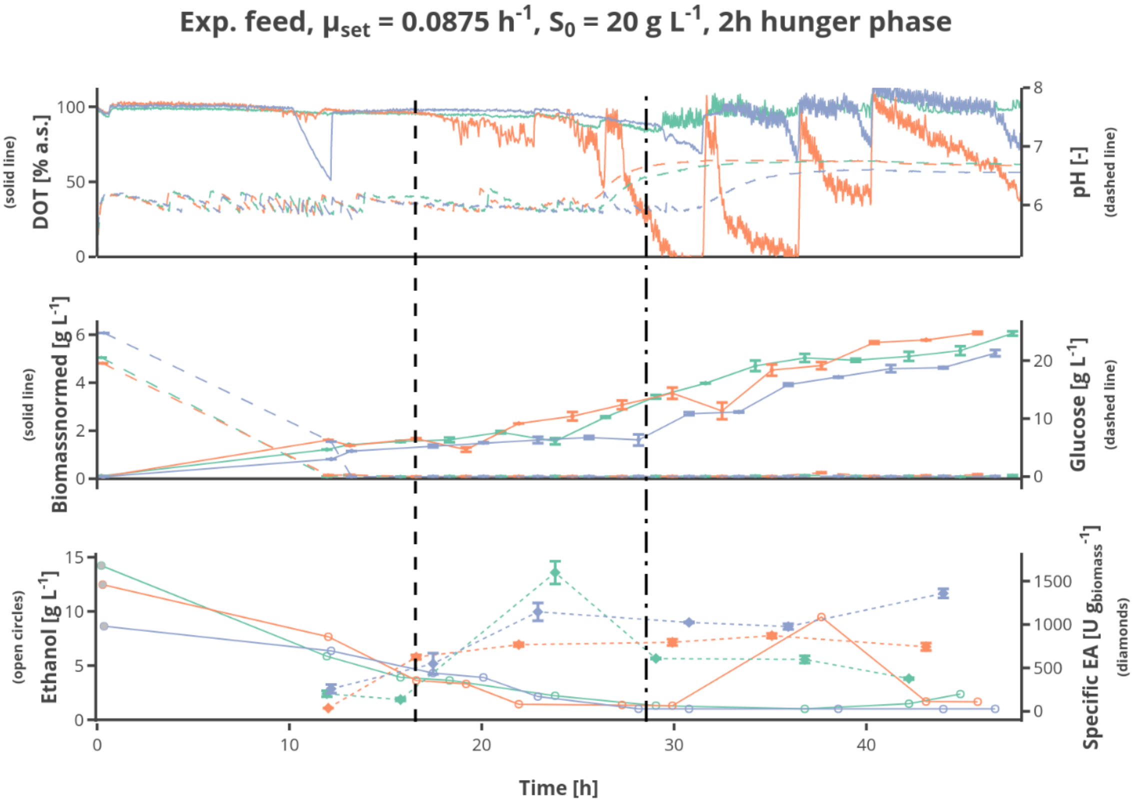
Exp. feed, μset = 0.0875 h-1, S0 = 20 g L-1, 2h hunger phase
(solid line)
DOT [% a.s.]
pH [-]
(dashed line)
(solid line)
Biomassnormed [g L-1]
Glucose [g L-1]
(dashed line)
(open circles)
Ethanol [g L-1]
Specific EA [U gbiomass-1]
(diamonds)
Time [h]

(**o**)

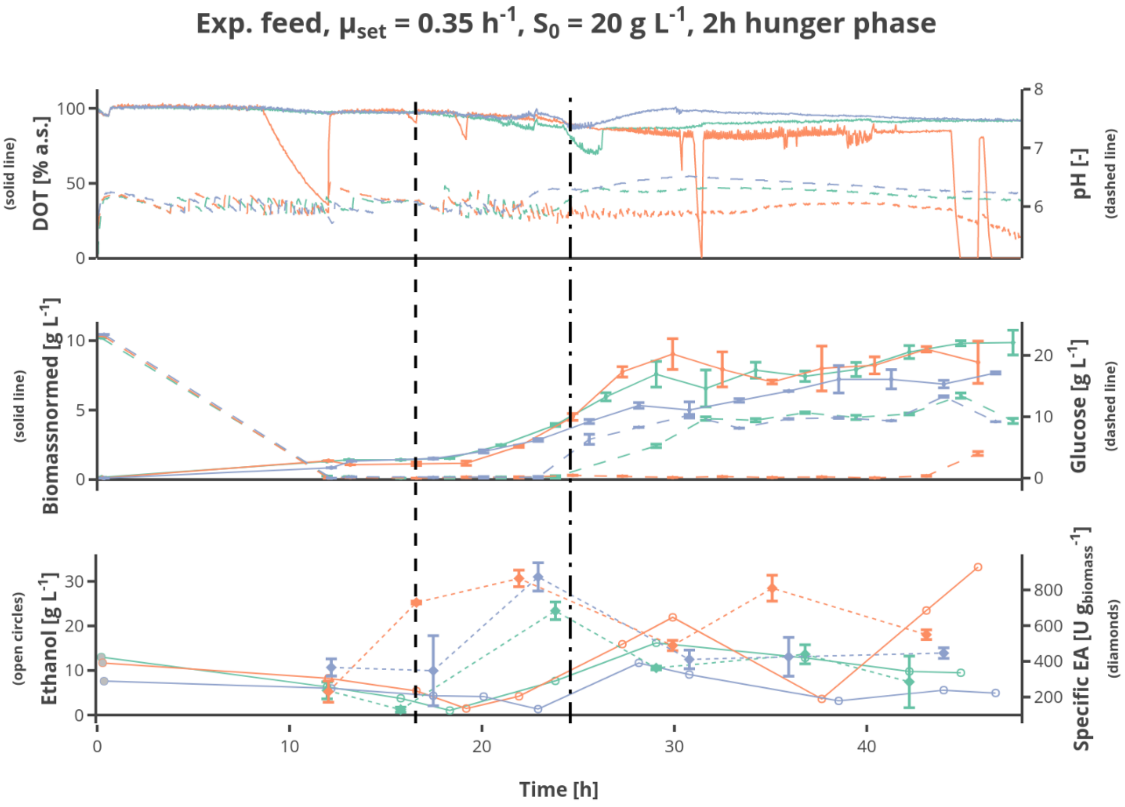

(**p**)

Figure S1. Cultivation data regarding growth at different applied cultivation conditions. (**a**) – (**p**) show the different cultivation conditions, details are given in the title. The DOT and pH, biomass and glucose concentration as well as ethanol concentration and specific enzyme activity are shown. The mean and standard deviation are shown regarding duplicate measurements for biomass and glucose concentration and regarding triplicate measurements for specific enzyme activity. The three MBR cultivations performed under the same conditions are shown in different colors, the start of the feed and the constant fed-batch phase are shown by the vertical dashed, respectively dashed-dotted line. Interactive versions of the plots can be found online [1].

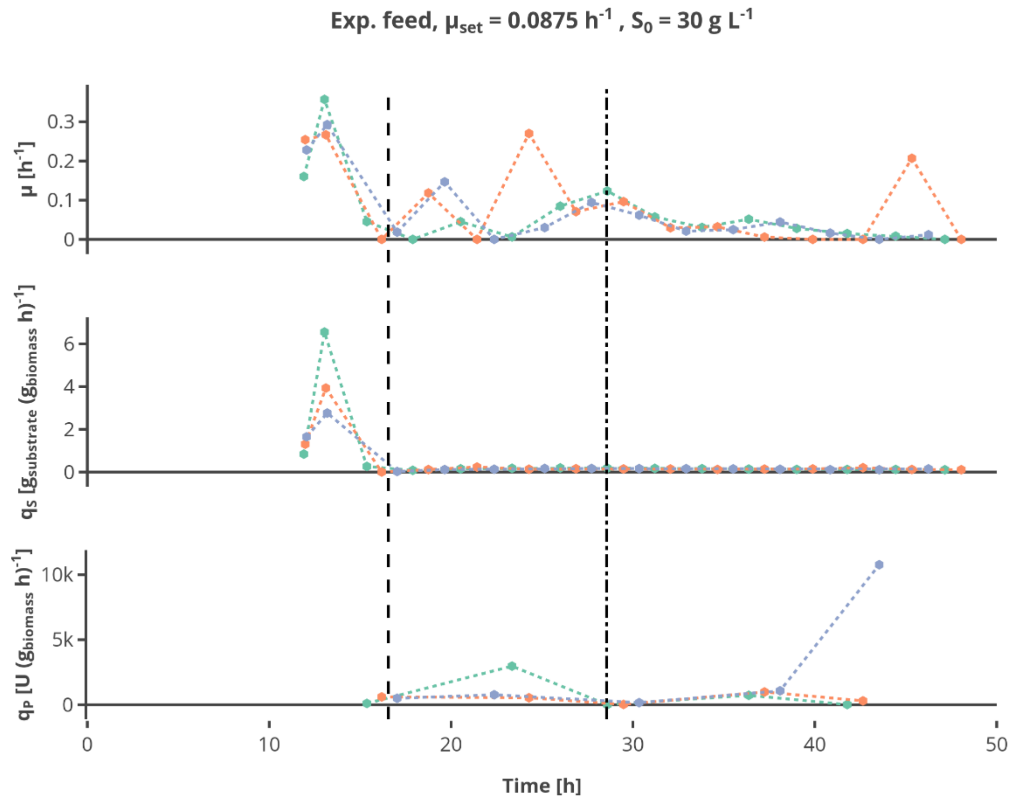
Exp. feed, μ_{set} = 0.0875 h^{-1} , S_0 = 30 g L^{-1}
μ [h^{-1}]
q_S [$g_{substrate}$ ($g_{biomass}$ h)$^{-1}$]
q_P [U ($g_{biomass}$ h)$^{-1}$]
Time [h]

(**a**)

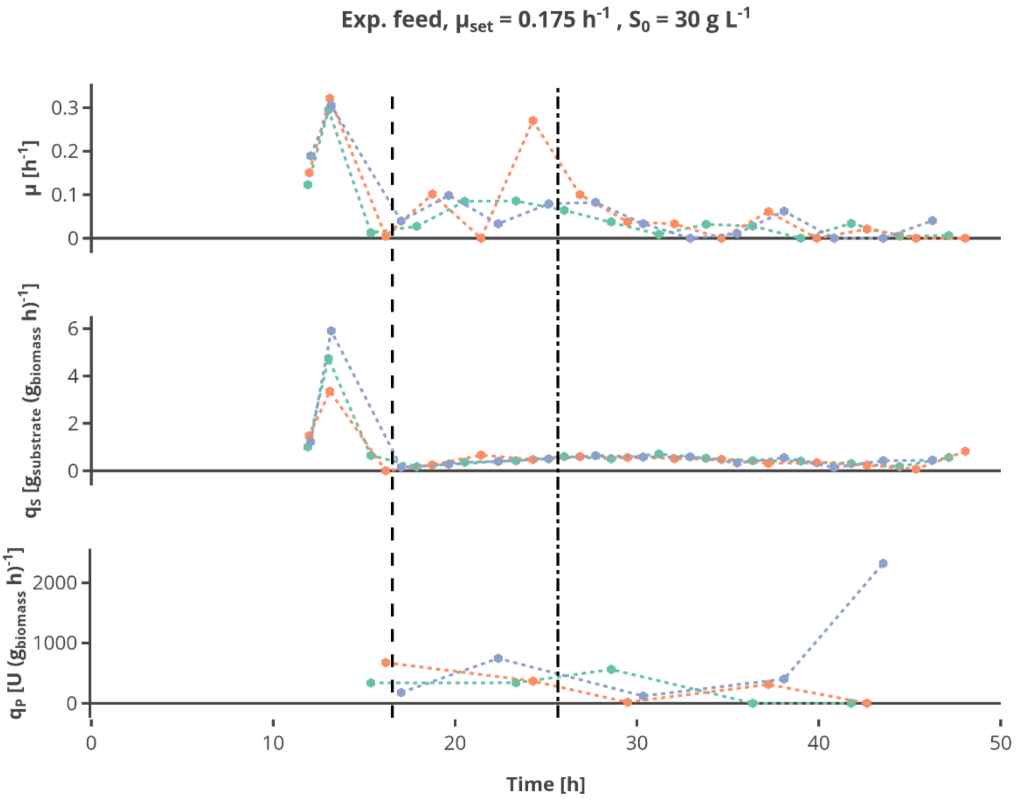
Exp. feed, μ_{set} = 0.175 h^{-1} , S_0 = 30 g L^{-1}
μ [h^{-1}]
q_S [$g_{substrate}$ ($g_{biomass}$ h)$^{-1}$]
q_P [U ($g_{biomass}$ h)$^{-1}$]
Time [h]

(**b**)

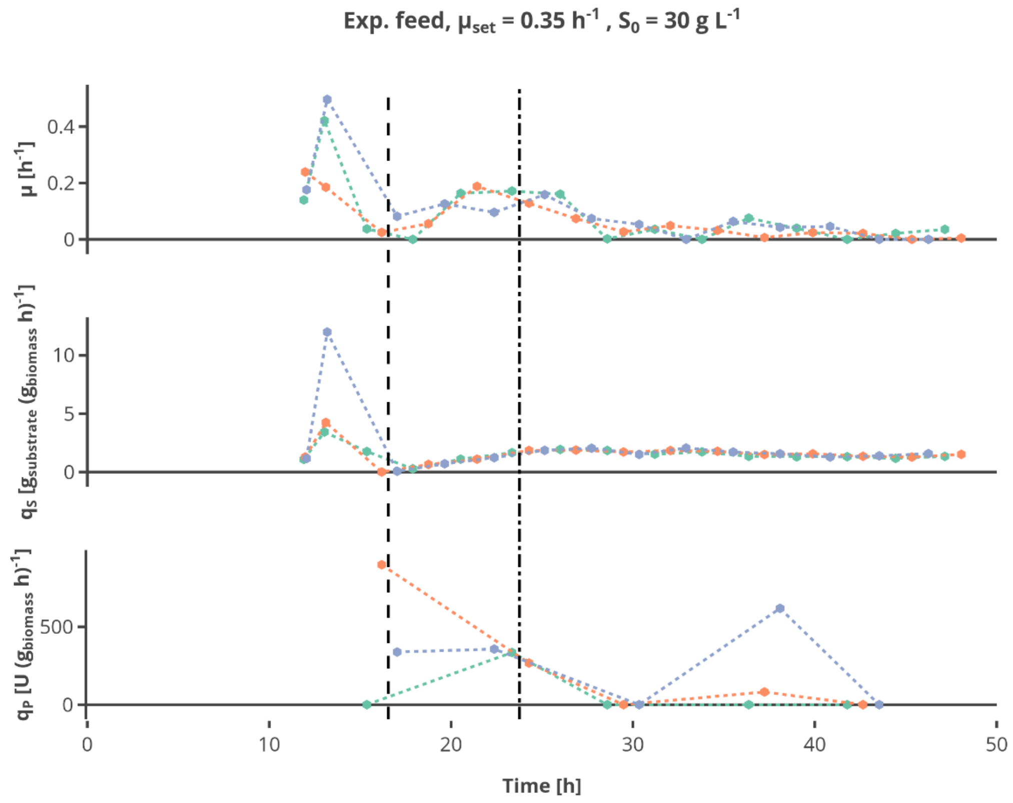
Exp. feed, μset = 0.35 h-1 , S0 = 30 g L-1
μ [h-1]
0.4
0.2
0
qS [gsubstrate (gbiomass h)-1]
10
5
0
qP [U (gbiomass h)-1]
500
0
0
10
20
30
40
50
Time [h]

(**c**)

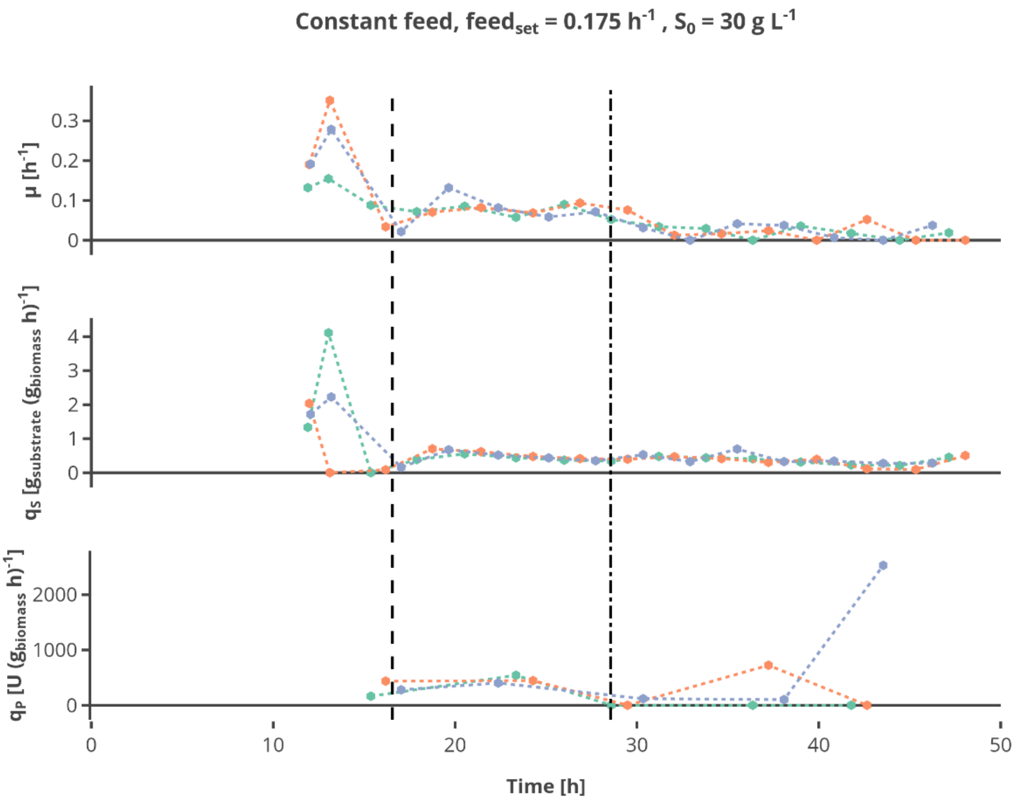
Constant feed, feedset = 0.175 h-1 , S0 = 30 g L-1
μ [h-1]
0.3
0.2
0.1
0
qS [gsubstrate (gbiomass h)-1]
4
3
2
1
0
qP [U (gbiomass h)-1]
2000
1000
0
0
10
20
30
40
50
Time [h]

(**d**)

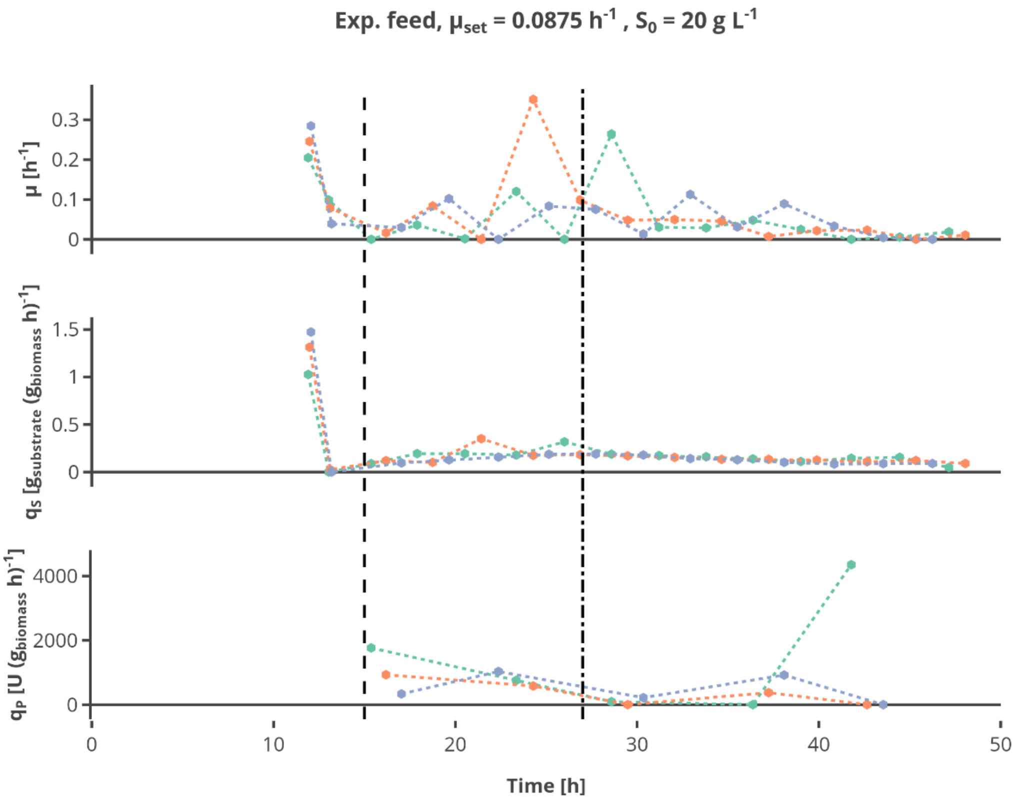
Exp. feed, μset = 0.0875 h-1 , S0 = 20 g L-1
μ [h-1]
qS [gsubstrate (gbiomass h)-1]
qP [U (gbiomass h)-1]
Time [h]

(**e**)

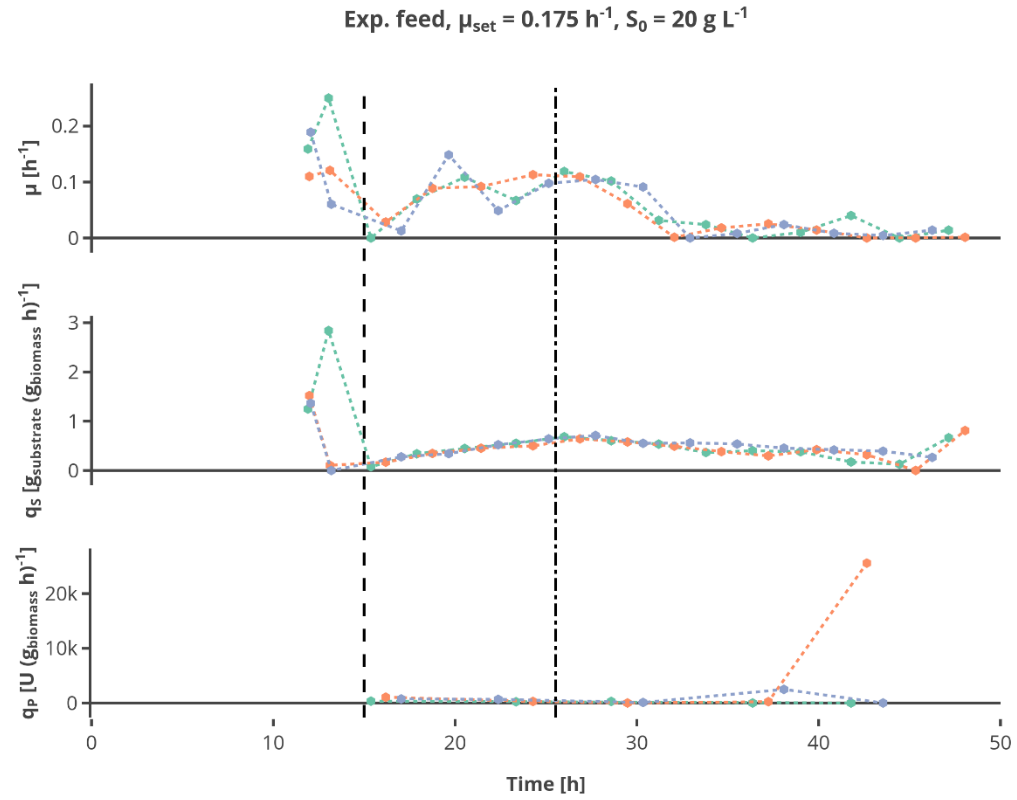
Exp. feed, μset = 0.175 h-1, S0 = 20 g L-1
μ [h-1]
qS [gsubstrate (gbiomass h)-1]
qP [U (gbiomass h)-1]
Time [h]

(**f**)

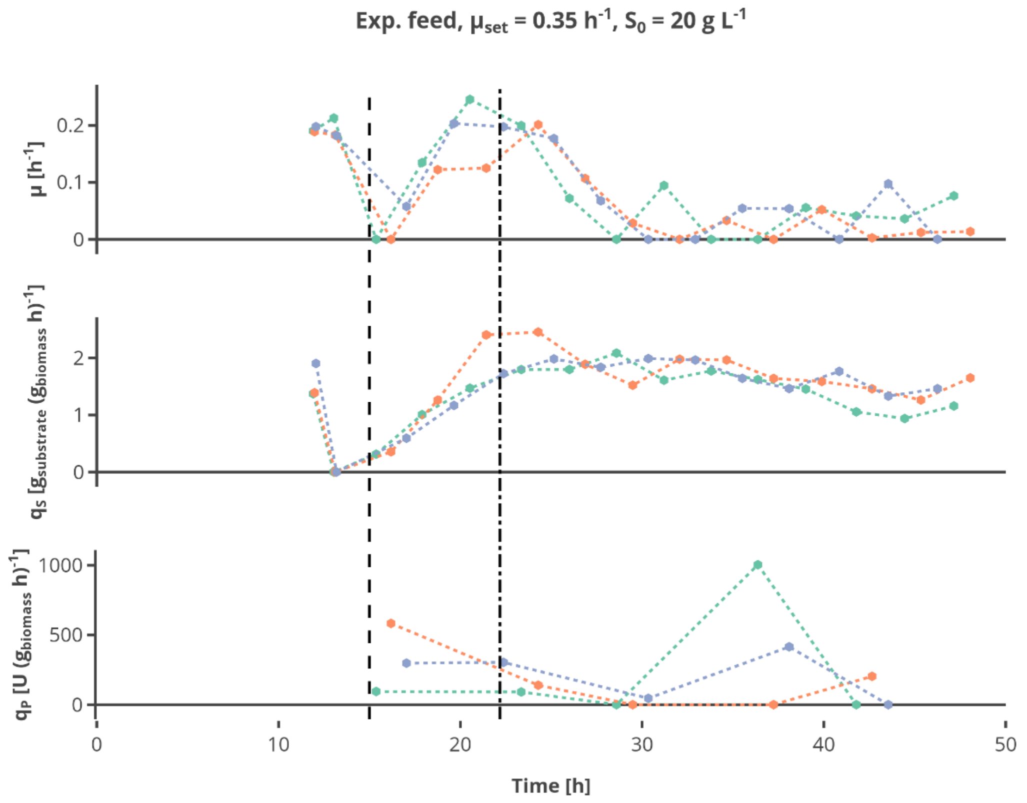
Exp. feed, μset = 0.35 h-1, S0 = 20 g L-1
μ [h-1]
qS [gsubstrate (gbiomass h)-1]
qP [U (gbiomass h)-1]
Time [h]

(**g**)

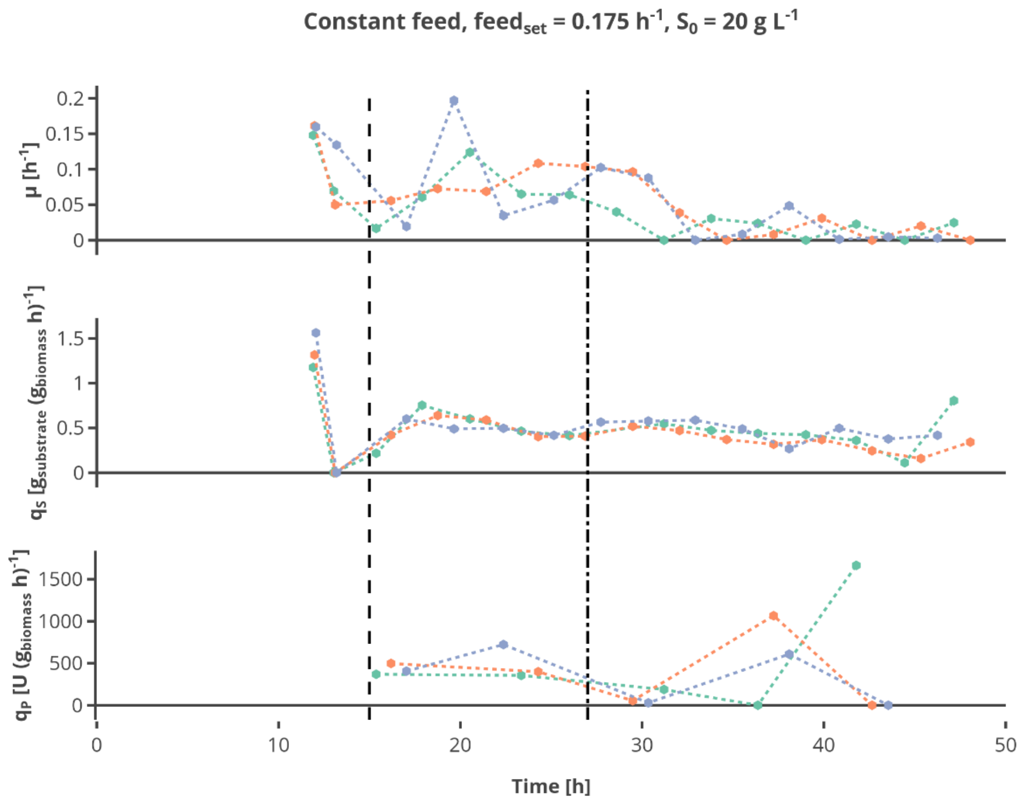
Constant feed, feedset = 0.175 h-1, S0 = 20 g L-1
μ [h-1]
qS [gsubstrate (gbiomass h)-1]
qP [U (gbiomass h)-1]
Time [h]

(**h**)

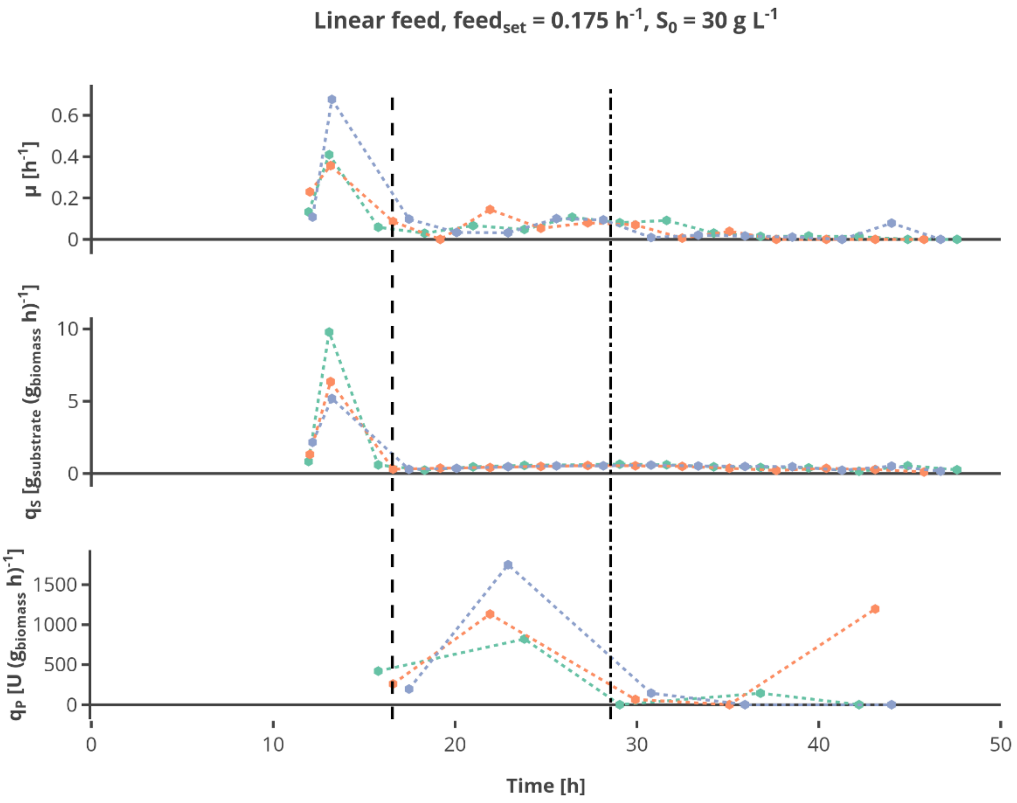
Linear feed, $feed_{set}$ = 0.175 h^{-1}, S_0 = 30 g L^{-1}
μ [h^{-1}]
q_S [$g_{substrate}$ ($g_{biomass}$ h)$^{-1}$]
q_P [U ($g_{biomass}$ h)$^{-1}$]
Time [h]

(**i**)

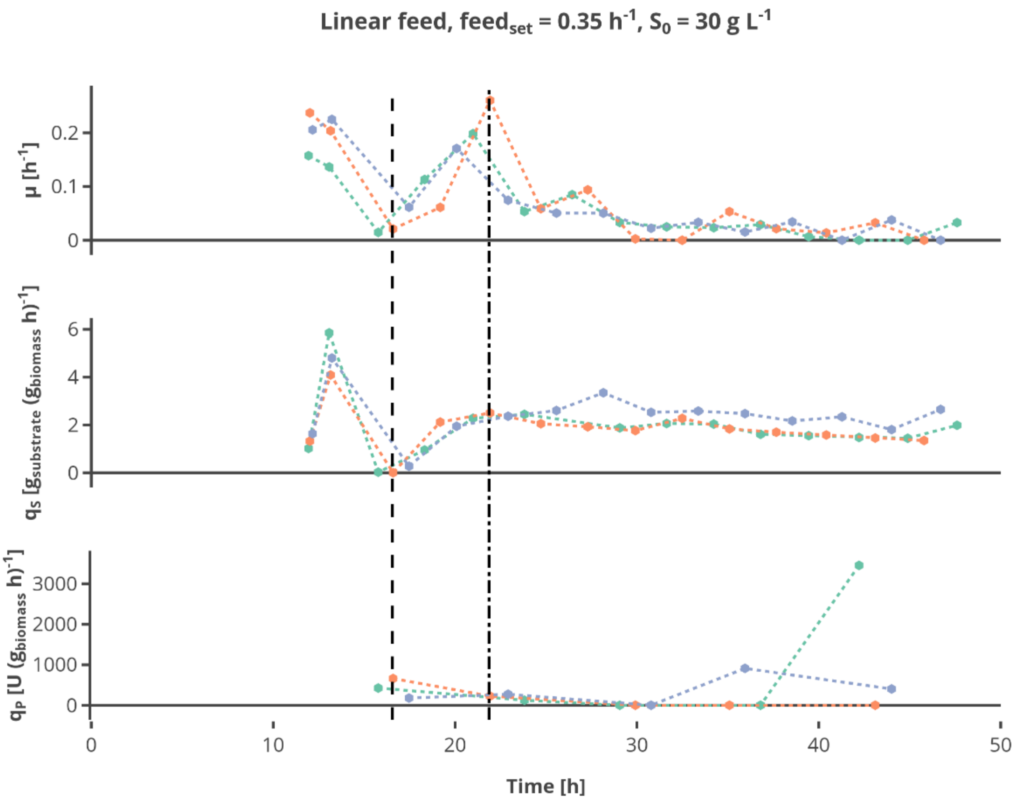
Linear feed, $feed_{set}$ = 0.35 h^{-1}, S_0 = 30 g L^{-1}
μ [h^{-1}]
q_S [$g_{substrate}$ ($g_{biomass}$ h)$^{-1}$]
q_P [U ($g_{biomass}$ h)$^{-1}$]
Time [h]

(**j**)

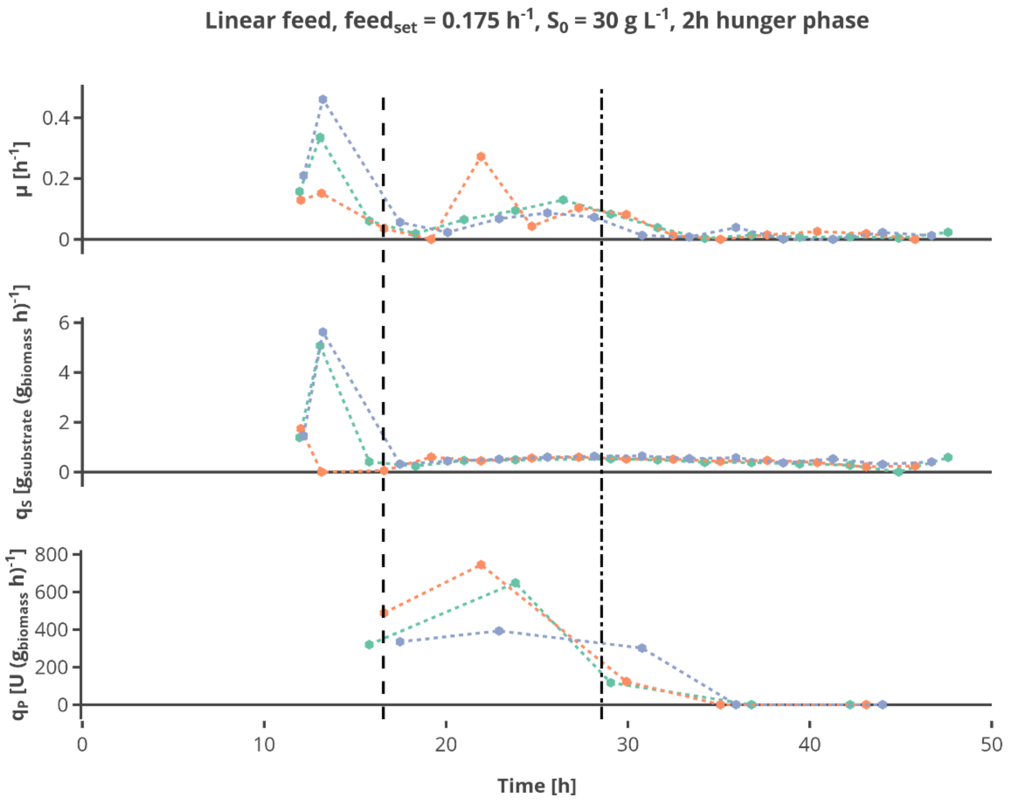
Linear feed, feed$_{set}$ = 0.175 h^{-1}, S$_0$ = 30 g L^{-1}, 2h hunger phase
μ [h^{-1}]
q$_S$ [g$_{substrate}$ (g$_{biomass}$ h)$^{-1}$]
q$_P$ [U (g$_{biomass}$ h)$^{-1}$]
Time [h]

(**k**)

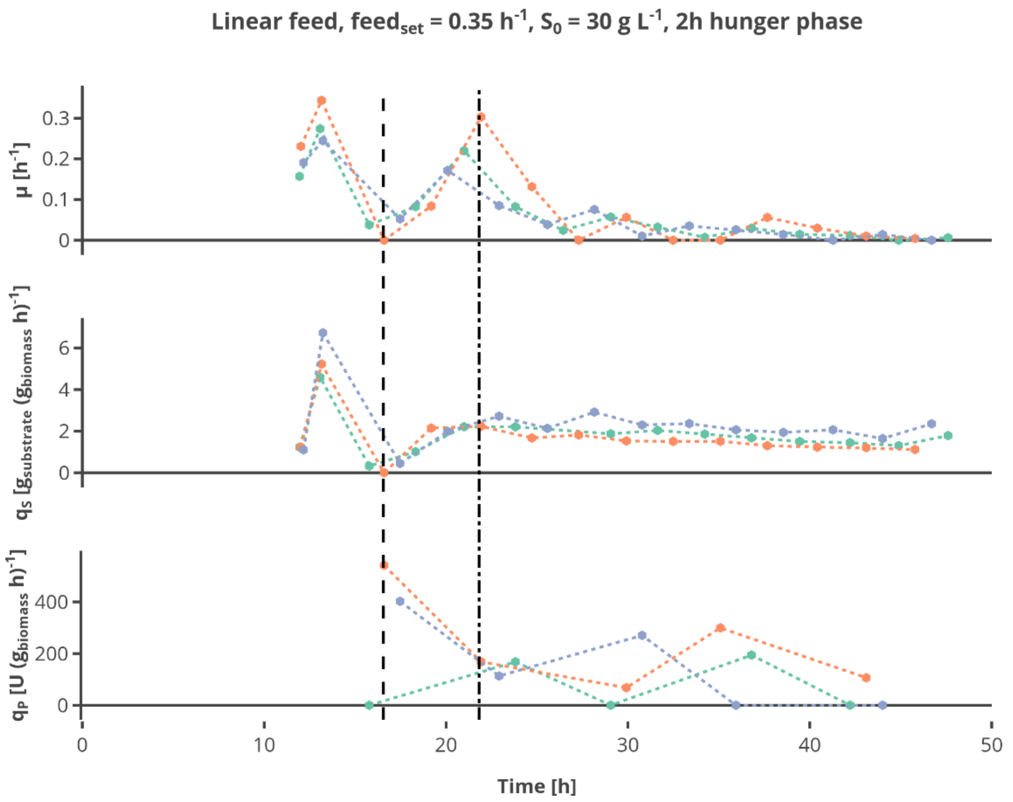
Linear feed, feed$_{set}$ = 0.35 h^{-1}, S$_0$ = 30 g L^{-1}, 2h hunger phase
μ [h^{-1}]
q$_S$ [g$_{substrate}$ (g$_{biomass}$ h)$^{-1}$]
q$_P$ [U (g$_{biomass}$ h)$^{-1}$]
Time [h]

(**l**)

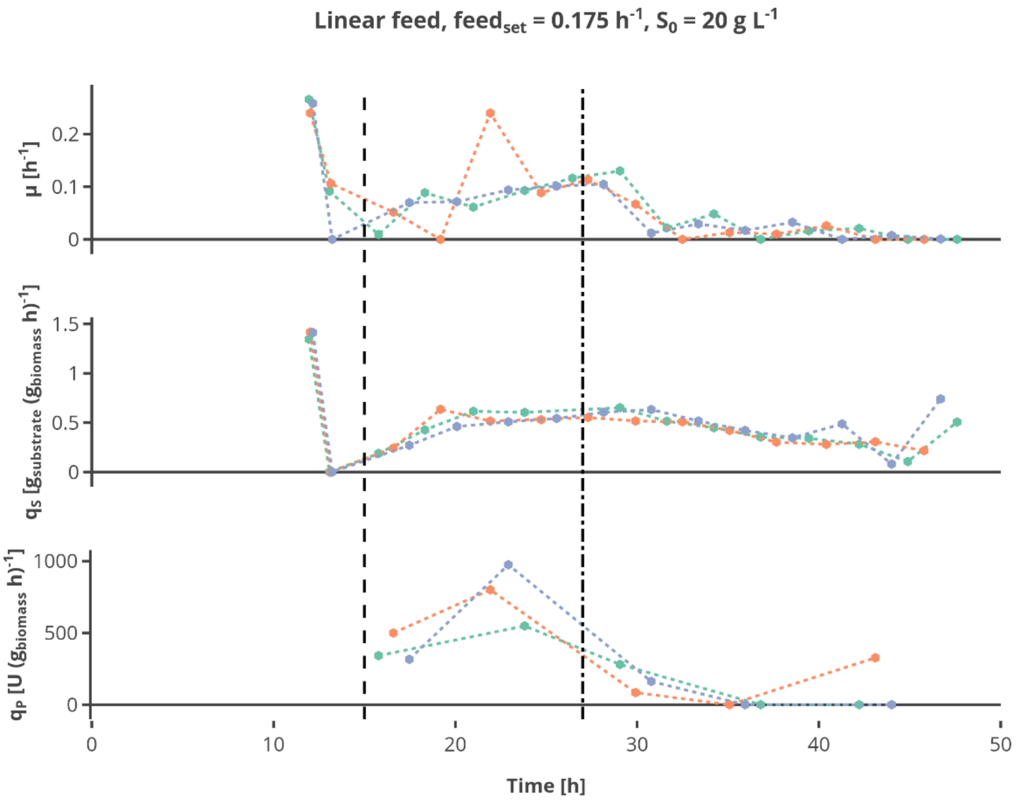

Linear feed, feedset = 0.175 h-1, S0 = 20 g L-1
μ [h-1]
qS [gsubstrate (gbiomass h)-1]
qP [U (gbiomass h)-1]
Time [h]

(m)

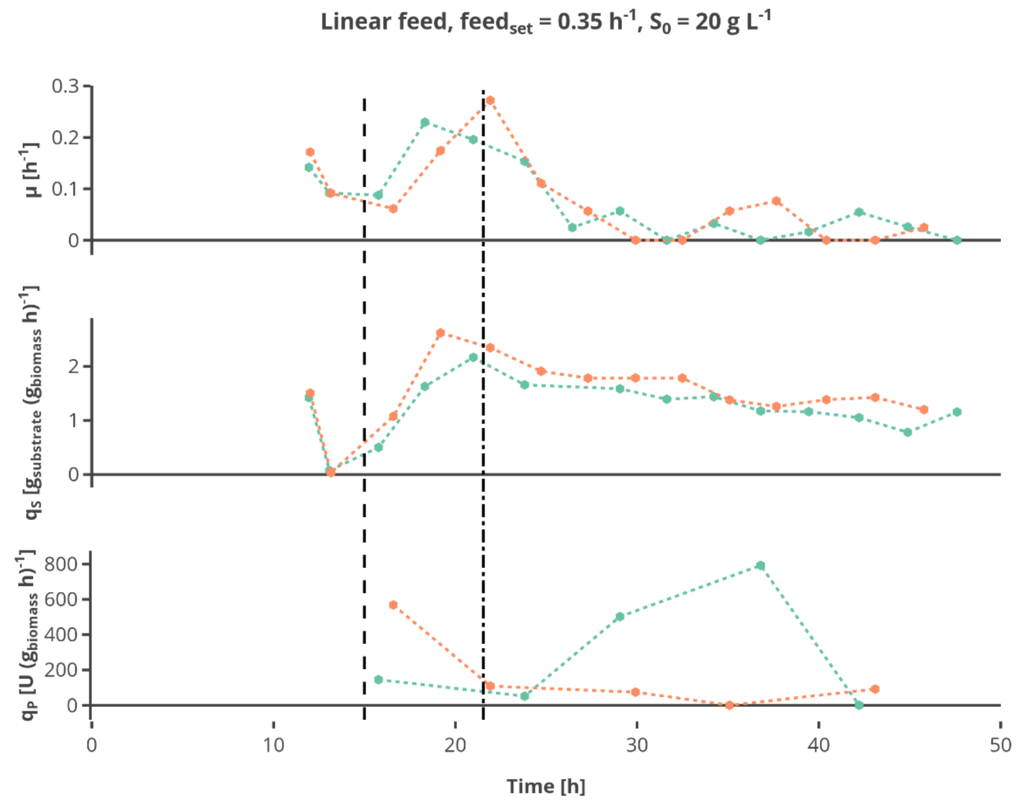

Linear feed, feedset = 0.35 h-1, S0 = 20 g L-1
μ [h-1]
qS [gsubstrate (gbiomass h)-1]
qP [U (gbiomass h)-1]
Time [h]

(n)

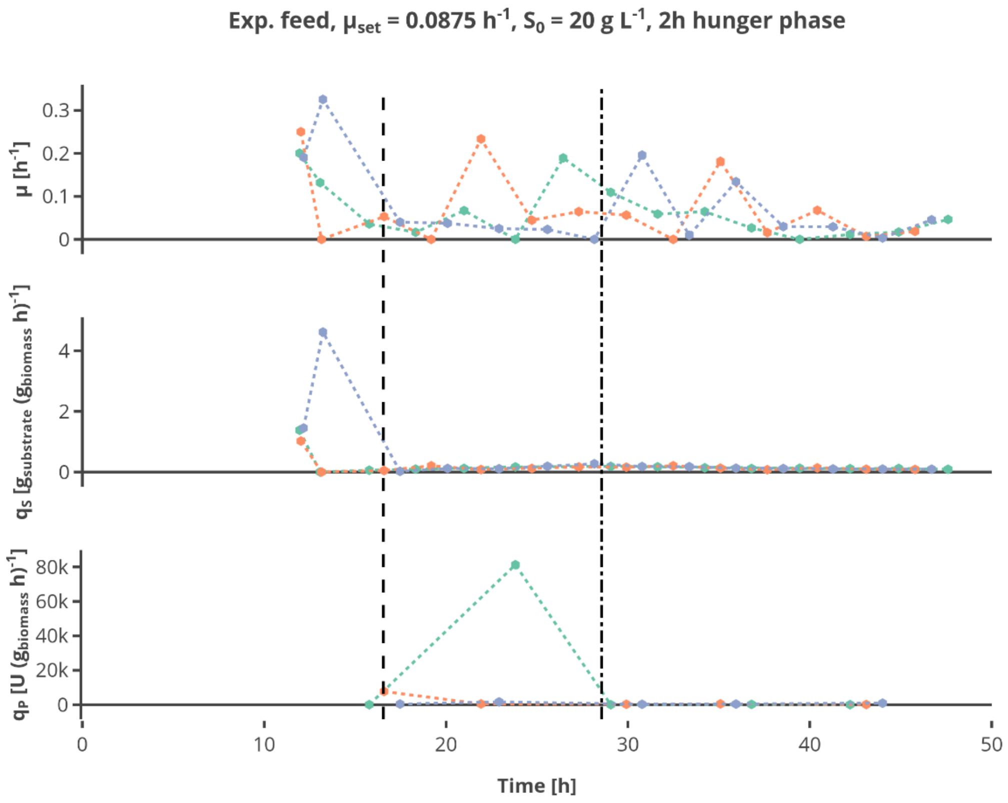

(**o**)

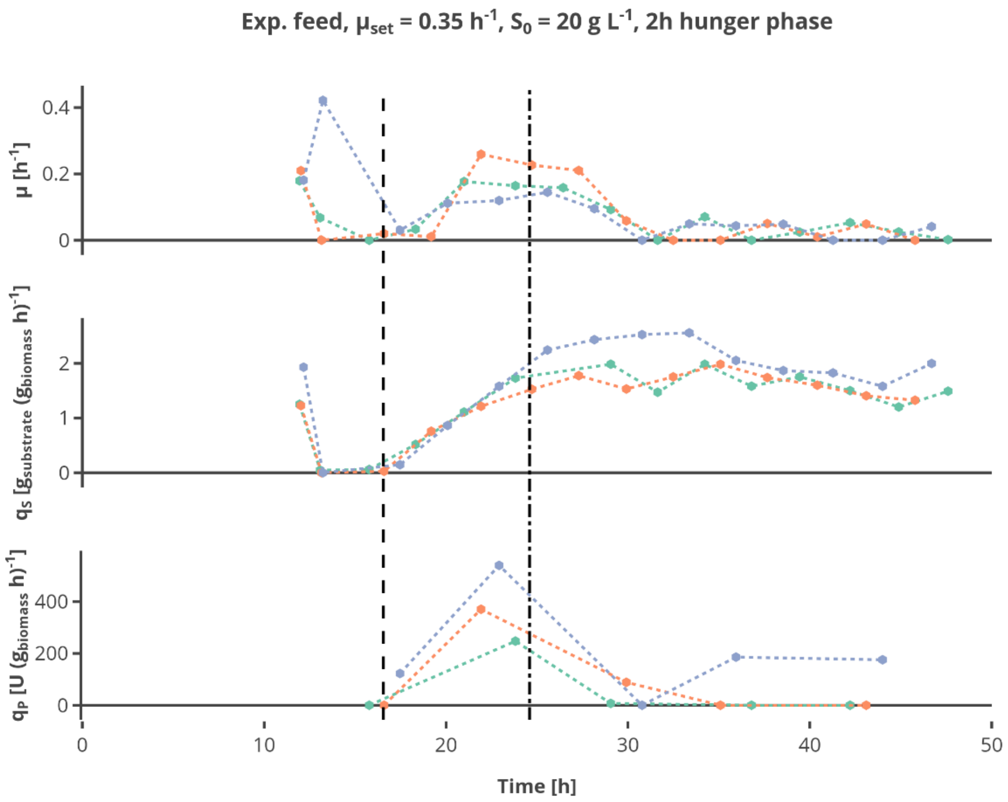

(**p**)

Figure S2. Specific rates for growth μ, substrate consumption q_S and product formation q_P regarding different applied cultivation conditions. (**a**) – (**p**) show the different cultivation conditions, details are given in the title. The rates are calculated using the mean of the respective measurements. The three

MBR cultivations performed under the same conditions are shown in different colors, the start of the feed and the constant fed-batch phase are shown by the vertical dashed, respectively dashed-dotted line. Interactive versions of the plots can be found online [1].

Table S1 shows the layout of the experimental conditions in the 2mag mini-bioreactor system. The replicates were distributed evenly in the mini-bioreactors, so that data for one replicate of each condition is obtained every 40 minutes.

Table S1. Layout of experimental conditions. The column and rows represent the layout of the bioreactor 48 fermentation system.

Row	Column 1	Column 2	Column 3	Column 4	Column 5	Column 6
A	1	9	1	9	1	9
B	2	10	2	10	2	10
C	3	11	3	11	3	11
D	4	12	4	12	4	12
E	5	13	5	13	5	13
F	6	14	6	14	6	14
G	7	15	7	15	7	15
H	8	16	8	16	8	16

Table S2 shows the volumetric and specific enzyme activity after 23 h and 37 h cultivation time, which are timepoints, which represent around one third and two thirds of fed-batch time. In Table S3 measurements of lactate, acetate, ammonia and phosphate are displayed after a cultivation time of 32 h. This time is critical as the cultivations have entered a growth arrest.

Table S2. Volumetric and specific enzyme activity (EA) after 23 h and 37 h cultivation time. These timepoints were chosen as they represent around one third and two thirds of fed-batch time. The MBR number and the design number are given. The mean enzyme activity with standard deviation is shown for each sample. Field underlined with red represent the four highest activities.

Bioreactor	Design	Volumetric EA [U mL^{-1}]		Specific EA [U $g_{biomass}^{-1}$]	
		23 h	37 h	23 h	37 h
1	1	3.86 ± 0.07	9.09 ± 0.58	1511.93 ± 27.16	1439.72 ± 91.30
2	2	2.31 ± 0.10	2.02 ± 0.44	681.20 ± 30.20	380.51 ± 82.49
3	3	4.04 ± 0.09	2.95 ± 0.50	887.53 ± 18.72	317.03 ± 54.24
4	4	3.69 ± 0.14	3.14 ± 0.04	1022.96 ± 38.12	516.46 ± 6.18
5	5	2.99 ± 0.11	2.80 ± 0.38	1179.48 ± 41.54	527.84 ± 71.99
6	6	1.37 ± 0.09	1.99 ± 0.16	530.55 ± 34.10	381.81 ± 30.73
7	7	1.96 ± 1.36	3.59 ± 0.17	313.62 ± 217.89	473.76 ± 22.71
8	8	2.43 ± 0.10	1.24 ± 0.44	780.00 ± 33.37	264.15 ± 92.98
9	9	3.83 ± 0.26	4.80 ± 1.63	1425.70 ± 96.15	766.98 ± 260.01
10	10	2.14 ± 0.30	1.97 ± 0.05	445.63 ± 61.59	246.45 ± 6.53
11	11	4.08 ± 0.27	3.78 ± 0.20	1296.78 ± 84.78	596.79 ± 31.12
12	12	2.78 ± 0.28	3.26 ± 0.16	506.52 ± 50.54	401.69 ± 19.29
13	13	3.30 ± 0.22	3.92 ± 0.16	1213.37 ± 79.58	654.24 ± 27.34
14	14	1.87 ± 0.12	4.82 ± 0.14	243.90 ± 15.11	498.55 ± 14.02
15	15	2.49 ± 0.20	3.01 ± 0.21	1598.30 ± 130.51	597.52 ± 42.11
16	16	2.68 ± 0.19	3.27 ± 0.39	682.72 ± 49.25	439.41 ± 53.07

17	1	4.08 ± 0.16	7.82 ± 0.77	1239.80 ± 49.81	1293.78 ± 127.11
18	2	3.55 ± 0.21	4.52 ± 0.32	1037.19 ± 60.93	800.60 ± 56.18
19	3	4.64 ± 0.12	2.52 ± 0.78	901.89 ± 24.24	301.14 ± 93.18
20	4	3.79 ± 0.23	5.88 ± 0.29	1038.37 ± 62.41	904.21 ± 44.83
21	5	2.69 ± 0.22	4.17 ± 0.07	1161.46 ± 96.59	944.59 ± 16.28
22	6	2.71 ± 0.04	2.55 ± 0.26	902.84 ± 12.17	486.76 ± 50.37
23	7	2.49 ± 0.14	0.64 ± 0.09	499.66 ± 27.62	85.34 ± 11.68
24	8	3.08 ± 0.09	5.97 ± 0.50	978.01 ± 29.16	1008.50 ± 83.65
25	9	3.49 ± 0.05	3.27 ± 0.11	1183.46 ± 17.86	575.29 ± 20.14
26	10	2.46 ± 0.11	1.87 ± 0.06	551.14 ± 25.59	249.60 ± 8.23
27	11	2.74 ± 0.23	3.80 ± 0.06	964.20 ± 79.43	708.77 ± 11.31
28	12	2.51 ± 0.07	2.78 ± 0.14	469.10 ± 13.44	347.36 ± 17.23
29	13	2.87 ± 0.14	1.82 ± 0.20	1106.48 ± 52.71	336.29 ± 36.31
30	14	1.86 ± 0.21	1.31 ± 0.23	376.22 ± 42.09	160.72 ± 28.10
31	15	1.77 ± 0.05	3.94 ± 0.12	768.04 ± 21.91	871.46 ± 26.36
32	16	2.07 ± 0.11	5.69 ± 0.51	864.04 ± 46.39	809.50 ± 72.49
33	1	2.75 ± 0.10	8.59 ± 1.56	1013.47 ± 37.09	1540.04 ± 278.77
34	2	2.26 ± 0.17	4.35 ± 0.02	803.76 ± 59.15	794.22 ± 4.37
35	3	2.39 ± 0.02	5.48 ± 1.18	697.28 ± 6.71	623.32 ± 134.64
36	4	2.10 ± 0.03	3.29 ± 0.82	649.15 ± 9.62	556.43 ± 138.68
37	5	1.48 ± 0.11	10.35 ± 0.83	861.37 ± 63.21	2086.74 ± 168.29
38	6	2.10 ± 0.04	6.32 ± 0.06	921.68 ± 19.30	1192.31 ± 11.97
39	7	2.67 ± 0.10	6.18 ± 0.67	687.26 ± 26.94	733.94 ± 79.11
40	8	2.57 ± 0.19	4.07 ± 0.94	1059.85 ± 79.24	786.50 ± 180.89
41	9	3.35 ± 0.03	3.41 ± 0.07	1224.38 ± 10.38	664.71 ± 13.49
42	10	2.20 ± 0.42	3.24 ± 0.24	606.34 ± 116.23	570.79 ± 42.08
43	11	2.03 ± 0.22	3.20 ± 0.05	753.06 ± 81.23	668.29 ± 9.64
44	12	2.01 ± 0.23	3.46 ± 0.15	502.19 ± 57.47	534.62 ± 22.55
45	13	3.00 ± 0.06	2.92 ± 0.07	1228.30 ± 24.89	598.52 ± 13.64
46 [1]	14	0.00 ± 0.00	0.08 ± 0.13	0.00 ± 0.00	1713.66 ± 2862.79
47	15	1.85 ± 0.17	3.82 ± 0.13	1145.46 ± 103.14	975.74 ± 32.73
48	16	2.48 ± 0.23	2.71 ± 0.69	872.97 ± 79.35	425.31 ± 108.56

[1] Bioreactor 46 was identified as outlier by PCA.

Table S3. Acetate, lactate, ammonia and phosphate concentration at ~32 h cultivation time. As mini-bioreactor 46 was identified as outlier, no measurements were obtained for this cultivation.

Bioreactor	Design	Acetate [mg L^{-1}]	Lactate [mg L^{-1}]	Ammonia [mmol L^{-1}]	Phosphate [mmol L^{-1}]
1	1	363.55	< 4.00	49.5	1143.77
2	2	59.35	< 4.00	47.84	874.8
3	3	1444.35	< 4.00	71.09	730.03
4	4	74.14	< 4.00	45.32	811.95

5	5	1881.52	< 4.00	60.4	1073.73
6	6	70.54	< 4.00	46.35	794.51
7	7	2175.29	< 4.00	63.46	471.07
8	8	75.21	< 4.00	32.48	871.37
9	9	284.06	< 4.00	46.84	832.36
10	10	2075.85	< 4.00	59.25	846.75
11	11	47.68	< 4.00	47.89	759.35
12	12	110.72	< 4.00	58.09	782.03
13	13	2237.42	< 4.00	40.05	713.77
14	14	307.60	< 4.00	67.28	467.86
15	15	89.26	< 4.00	63.24	1085.11
16	16	1895.83	< 40.00	52.24	704.91
17	1	101.66	< 40.00	60.33	1178.9
18	2	< 100.39	< 40.00	50.85	881.81
19	3	820.93	< 40.00	57.78	826.67
20	4	< 100.39	< 40.00	42.83	787.8
21	5	< 100.39	< 40.00	66.72	1182.69
22	6	< 100.39	< 40.00	43.37	866.09
23	7	1867.63	< 40.00	60.52	826.74
24	8	< 100.39	< 40.00	37.07	712.85
25	9	105.05	< 40.00	37.61	918.61
26	10	1180.18	< 40.00	58.12	833.05
27	11	< 100.39	< 40.00	43.27	826.56
28	12	2298.01	< 40.00	61.46	615.06
29	13	< 100.39	< 40.00	44.49	776.06
30	14	< 100.39	< 40.00	43.96	777.33
31	15	< 100.39	< 40.00	64.97	1211.51
32	16	2182.68	< 40.00	71.83	391.01
33	1	102.05	< 40.00	77.9	1185.49
34	2	< 100.39	< 40.00	48.84	841.63
35	3	1471.98	< 40.00	69.61	905.6
36	4	106.78	< 40.00	49.9	883.23
37	5	< 100.39	< 40.00	76.34	1276.58
38	6	135.60	< 40.00	51.2	839.84
39	7	2453.30	< 40.00	72.73	706.1
40	8	101.09	< 40.00	44.32	816.43
41	9	106.15	< 40.00	54.77	885.61
42	10	715.61	< 40.00	58.27	966.07
43	11	309.98	< 40.00	51.1	882.59
44	12	761.89	< 40.00	59.38	963.25
45	13	< 100.39	< 40.00	48.75	824.27
46 [1]	14	-	-	-	-

47	15	< 100.39	< 40.00	82.47	1296.95
48	16	865.10	< 40.00	55.5	960.43

[1] Bioreactor 46 was identified as outlier by PCA.

References

1. Experimental Results Sawatzki_2018. Available online: https://www.tu-berlin.de/?200026 (accessed November 19, 2018).
